# Beyond Bioactive Glass Composition: Using Morphology to Improve In Vitro and In Vivo Performance

**DOI:** 10.1002/adhm.202502591

**Published:** 2025-09-16

**Authors:** Meixin Su, Mert Ergin, Diana Horkavcová, Victoria Horbert, Georg Matziolis, Delia S. Brauer

**Affiliations:** ^1^ Otto Schott Institute of Materials Research Faculty of Chemistry and Earth Sciences Friedrich Schiller University Lessingstr. 12 (AWZ) 07743 Jena Germany; ^2^ Orthopaedic Department Jena University Hospital Friedrich Schiller University Campus Eisenberg Klosterlausnitzer Str. 81 07607 Eisenberg Germany; ^3^ Laboratory of Chemistry and Technology of Glasses Department of Glass and Ceramics University of Chemistry and Technology Technická 5 Prague 6 166 28 Czech Republic

**Keywords:** bioactive glass, microbes, morphology, osteoblasts

## Abstract

Compared to compositional functionalization with therapeutic ions, changing bioactive glass (BG) morphology, i.e., the size, shape, and surface structure of BG specimens, is typically not considered as an influential factor for improving performance. However, it has a large influence on the outcome of various experiments, particularly those studies where the material comes in contact with aqueous solutions, including acellular immersion studies, in vitro experiments using cells or bacteria, and even animal experiments and clinical studies. One major aspect is the surface area to volume ratio, the rate at which ions are released from the glass and surface layers, including apatite layers, forming, can be tailored to subsequently influence the behavior of cells and bacteria. By varying surface roughness or patterning, the orientation and alignment of cells can be controlled or the adhesion of bacteria can be prevented, both in vitro and in vivo. Taken together, this review shows that BG morphology is an important parameter to consider when designing experiments or developing clinical products.

## Graphical abstract

Bioactive glasses can easily be shaped into granules, spheres, discs, fibers, or three‐dimensional scaffolds. The resulting morphology not only affects handling properties; it has a direct influence on various glass properties, including results of acellular immersion experiments or in vitro studies with cells or bacteria, but also on in vivo performance

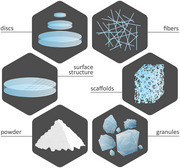

## 1 Introduction

Bioactive glasses (BG) are successfully used to treat non‐load bearing site bone defects,^[^ ^1^ ^]^ and variation in composition and atomic‐scale structure with the subsequent changes in therapeutic properties^[^ ^2^ ^]^ is the accepted approach for tailoring BG properties to their clinical application. BG morphology, by contrast, seems to be considered for technical reasons mostly, such as ease of use. Its importance in BG performance, however, is reflected in how they have been used clinically, and how their morphology changed along the way, The first BG clinical use was for reconstruction of the ossicular chain to conduct sound from the tympanic membrane to the cochlea and restore hearing,^[^ ^3^ ^]^ and to this purpose a 45S5 solid piece prepared by casting was implanted into the inner ear. Nowadays, BG are used to regenerate, rather than replace, bone in non‐load bearing sites, and granules rather than solid pieces are used.^[^ ^4^ ^]^ Clearly, BG morphology and clinical application are closely connected, which leads to the question of how we can use BG morphology to improve BG performance, not only from an engineering perspective but also from a biomedical one. Morphology may control how well an implant works, how fast it degrades, releases ions, helps cells to adhere, proliferate, or differentiate, and how well it fights bacteria.

One great advantage of glassy materials is that they can easily be shaped at high temperatures, making changes to BG morphology simple. BG can be drawn into fibers or sintered into porous scaffolds; they can also be cast into blocks and cut into discs, crushed into granules, or milled into fine powder. Although the most well‐known BG, Bioglass 45S5 (composition in wt.%, 45 SiO_2_, 6 P_2_O_5_, 24.5 CaO, 24.5 Na_2_O) or BonAlive S53P4 (in wt.%, 53 SiO_2_, 4 P_2_O_5_, 20 CaO, 23 Na_2_O), crystallize easily at elevated temperatures and, therefore, are not perfectly suited for high temperature processing,^[^ ^5^, ^6^ ^]^ they are the most used BG compositions and have been processed into various shapes. On these, many studies have been published, and several of them compare different BG morphologies directly, i.e., either parallel in one experimental study or using identical experimental parameters. This makes analysis of the influence of BG morphology on the experimental outcome comparatively straightforward, as other influencing factors can be largely excluded.

Considering the vast amount of literature on 45S5 and S53P4, we have chosen to focus on these two compositions to identify routes for tailoring BG biomedical performance via their morphology.

## 2 Effects of BG Morphology During Acellular Immersion Experiments

**Figure 1:**
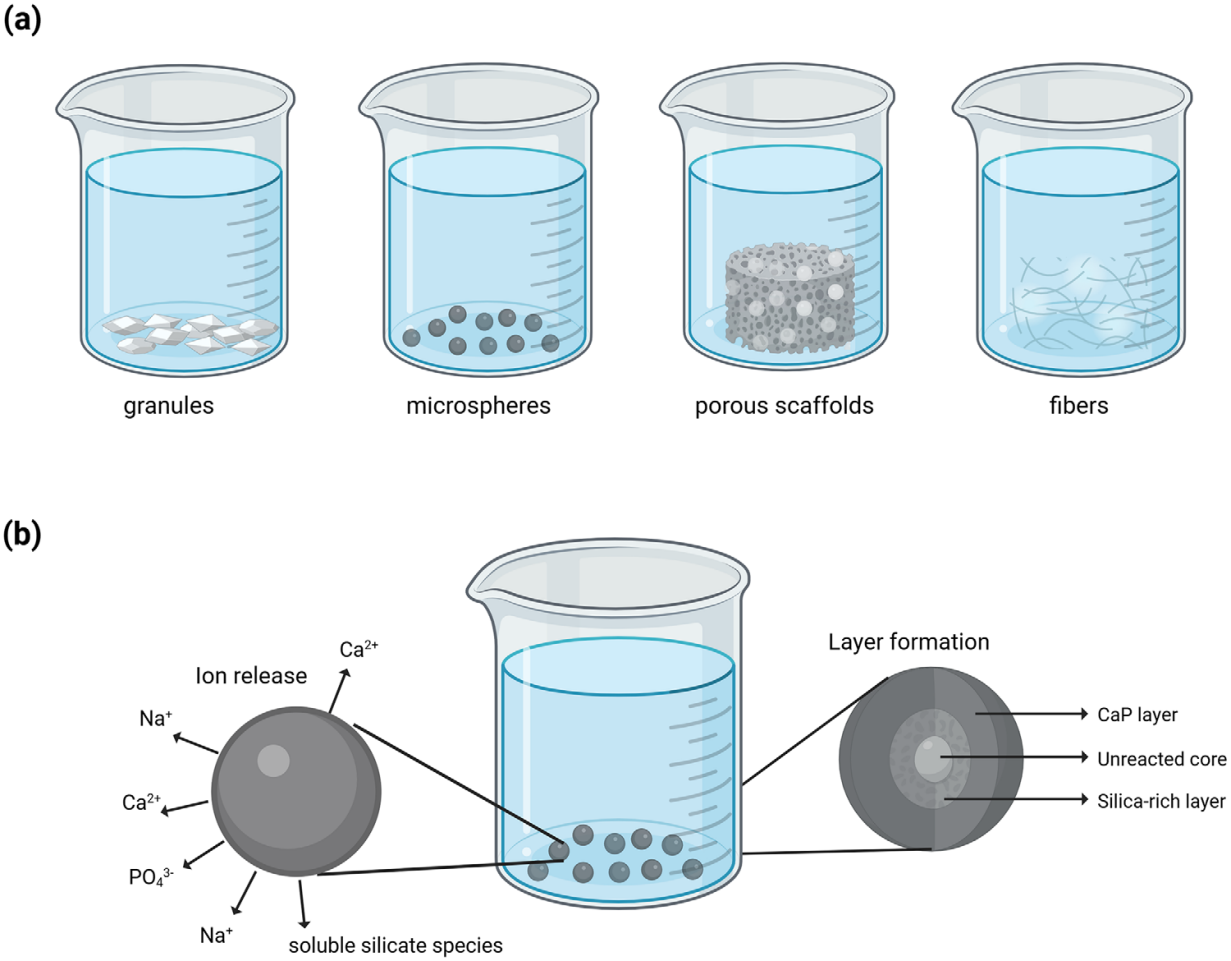
a) In acellular immersion experiments, BG of various morphologies are immersed in testing solutions such as simulated body fluid. b) During immersion, they release ions and form surface layers (calcium phosphate/CaP and silica‐rich layers), the details of which can provide information on the glass's reactivity. Figure created in BioRender. Horbert, V. (2025) https://BioRender.com/wue0cz7.

BG such as 45S5 or S53P4 have a low silica content compared to conventional silicate glasses,^[^ ^7^, ^8^ ^]^ resulting in distinct structural features at an atomic or molecular level. As a result, these materials are highly surface reactive, showing fast reactions in contact with aqueous solutions, resulting in the rapid release of ions. This, subsequently, causes mineralization of an apatite layer, typically a substituted hydroxycarbonate apatite, on the glass surface.^[^ ^9^ ^]^ Simple immersion tests in simulated physiological solutions, such as simulated body fluid (SBF), tris(hydroxymethyl)aminomethane‐HCl (Tris‐HCl) buffer solution, or acellular culture media, can give insight into how the material interacts with aqueous environments.^[^ ^9^ ^]^ These solutions are typically free of proteins, but some experiments with protein‐containing solutions have been performed.^[^ ^10^ ^]^ Experiments usually aim to investigate changes both in the sample, e.g., surface layer formation, and in the solution, such as changes in pH and ion concentration (**Figure** **1**).^[^ ^9^ ^]^ Here, we focus on the influence of BG morphology on these changes (Table S1, Supporting Information).

**Figure 2:**
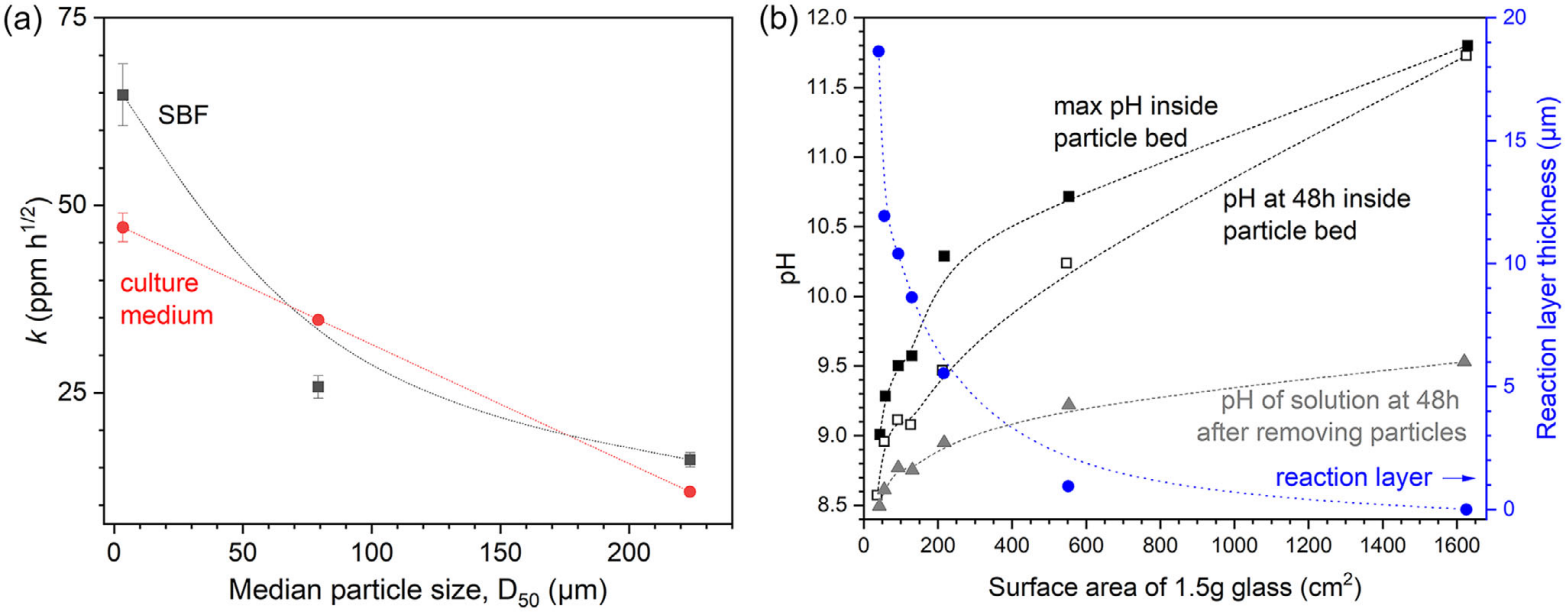
a) Initial rate of Si release from 45S5 immersed in either SBF or a‐MEM. *k* is the gradient of linear fitting applied to plots of c(Si) = *k* t^½^ at time points before dissolution reached a minimum. Graph plotted based on data presented by Sepulveda et al.^[^ ^11^ ^]^ b) pH and reaction layer thickness (right axis, blue) during immersion of 45S5 of various particle size ranges, plotted over the total surface area of 1.5 g of glass, pH inside particle bed at 48 h and maximum value and pH of solution at 48 h after removal of particles. Graphs plotted based on data presented by Zhang et al.^[^ ^12^ ^]^ Lines are visual guides.

Reactions during contact between BG and aqueous media strongly depend on the relative surface area. Results clearly demonstrate that a decrease in particle size or an increase in surface area to volume (SA/V) ratio results in faster reactions, as detailed in the following studies:

Work by Sepulveda et al.^[^ ^11^ ^]^ presented the dissolution characteristics of three particle size ranges of melt‐derived 45S5 in SBF or α‐MEM (Minimum Essential Medium) culture medium. Results showed that at 24 h, the pH of each immersion medium showed pronounced variation with particle size, with the fine powder (5 to 20 µm) showing the highest pH (between 8 and 8.5 in SBF) while medium‐sized (90–300 µm) and coarse (90–710 µm) particles showed a maximum pH of 7.5 and 8, respectively in SBF. Ion concentrations in solution also varied with particle size but were additionally affected by apatite precipitation, which consumes some ions from the solution (phosphate and calcium mostly) but not others (soluble silicate species). As a result, silicon concentrations in solution showed a clear trend, with the fine particles resulting in the highest concentrations and the coarse ones in the lowest. The authors also investigated the kinetics of silicon network dissolution and showed that the initial rate of Si release decreased with increasing particle size (**Figure** **2**a). Calcium concentrations showed the trend of the largest concentrations for smallest particle sizes at early time points only, and phosphate concentrations of the fine powders dropped very rapidly owing to apatite formation, remaining lowest of the three fractions tested. Both effects may result from apatite precipitation being fastest for the smallest particle size range; however, the authors apparently did not observe any differences in X‐ray diffraction (XRD) or Fourier‐transform infrared spectroscopy (FTIR) results between particle size ranges, suggesting that no pronounced differences in apatite precipitation existed. Unfortunately, the publication shows XRD and FTIR results for fine powders only.

Mačković et al.^[^ ^13^ ^]^ compared the in vitro reactivity of two particle size ranges by immersing flame‐sprayed nanoscale particles (20–60 nm; nBG) and melt‐derived microparticles (median diameter, D_50_, 10 µm; µBG) of 45S5 in SBF for up to seven days. The as‐prepared amorphous nBG particles were initially fused together, forming chain‐like aggregates prior to SBF treatment. Upon immersion in SBF, both hydroxyapatite (HA) and calcite phases formed, accompanied by a reduction in particle size and disruption of the chain‐like aggregation. By contrast, µBG showed only HA formation throughout the reaction process, with no evidence of calcite detected by either XRD or FTIR. FTIR analysis revealed that the characteristic apatite‐associated bands appeared at earlier time points and with higher intensity for nBG than for µBG. This is attributed to the significantly larger specific surface area of nBG, which enhances reactivity.

The effect of particle size is comparable for 45S5 and S53P4. Zhang et al.^[^ ^12^ ^]^ investigated a broad range of particle size fractions of 45S5 or S53P4 immersed in SBF and compared them to immersed glass plates. pH was measured inside the particle bed and, additionally, in the bulk solution at the end of the experiment after removing the glass particles. In either case, pH showed a pronounced increase with decreasing particle size for both BG compositions. Results were plotted as a function of total BG surface area, confirming that an increase in surface area resulted in an increased pH of the immersion medium (Figure 2b, shown for 45S5 only). The authors identified three types of reaction layers: silica‐rich, calcium phosphate‐rich, and a mixed layer of the two; layer thickness also varied with particle size, plates showed relatively even layers, their thickness varying with glass composition; 45S5 showed thicker layers than S53P4. For particles, maximum layer thickness decreased with relative surface area, with the smallest particle size range showing thin and sporadic layers only or none at all (Figure 2b). This suggests that the conditions created by the smallest particles, i.e., a pH above 11, impeded apatite formation. While apatite stability increases with pH increasing from 4 to about 9, it decreases for higher values. At pH 12, hydroxyapatite solubility is nearly identical to the one at pH 4,^[^ ^14^ ^]^ which may have prevented apatite mineralization for fine particles.

Is the SA/V ratio is kept constant, changes in the size of the BG specimens are expected to be negligible. Clupper et al.^[^ ^15^ ^]^ investigated apatite surface layer formation on 45S5 fibers of either 20 or 40 µm in diameter and 2 cm in length. During their immersion experiments in SBF, they kept the SA/V ratio constant for both fiber types, at 1 cm^2^ mL^−1^. Based on this constant SA/V ratio, the authors expected apatite layer formation, detected using FTIR only, to be comparable for both fiber types. The earlier appearance of the split band at around 600 cm^−1^ (corresponding to the absorption band of PO_4_^3−^ in apatite^[^ ^16^ ^]^) for the 40 µm fibers (two days compared to four days for the 20 µm fibers), the authors explained by the presence of a silane coupling agent present on the 20 µm fibers impeding layer formation.^[^ ^15^ ^]^

Changes in BG composition during the preparation of BG specimens may counteract changes in SA/V, Sinitsyna et al.^[^ ^17^ ^]^ investigated the dissolution behavior of flame‐sprayed commercial S53P4 microspheres across different size fractions. Microspheres ranging in size from 45 to 500 µm were immersed in either Tris‐HCl or SBF solutions (starting pH 7.4 at 37 °C) under dynamic or static conditions. Ion concentrations were measured using ICP‐OES, and pH was monitored over time. Results were surprising, as ion release from the largest microspheres was much higher than from the smaller fractions. One would expect the opposite trend, as smaller microspheres have a larger SA/V ratio, leading to faster ion release. Calculated normalized surface‐specific mass loss rate and surface layer formation (silica‐rich and CaP layers) showed the same unusual trend of larger spheres reacting faster than the smaller ones. Compositional analysis of the pristine (i.e., unreacted) spheres provided the explanation, as it showed that only the largest particles (300–500 µm) had a composition close to the nominal one. With decreasing particle size, composition deviated from the nominal one, and particles showed much reduced sodium and phosphate contents, resulting in much higher silica contents and, thus, a more polymerized silicate network. An increased silicate network polymerization, often described as the network connectivity, defined as the average number of bridging oxygens per silicon atom,^[^ ^8^, ^18^ ^]^ is known to reduce glass reactivity. The compositional changes during flame‐spraying meant that the smallest spheres in the study (45–90 µm) had an average network connectivity of 2.89 ± 0.06 rather than the normal network connectivity of 2.54 for S53P4.^[^ ^7^ ^]^ Thus, the experimental results do not actually reflect changes in glass morphology but in glass composition and structure.

In a follow‐up study, Sinitsyna et al.^[^ ^19^ ^]^ compared two particle sizes of flame‐sprayed S53P4 microspheres (45–90 or 90–125 µm) in dynamic dissolution studies for up to 72 h. Here, the composition of the microspheres was close to the nominal one, and no compositional differences with size were apparent. Therefore, unlike their previous study, no significant differences in ion release or surface layer formation with microsphere size were observed, most likely because the two size fractions were relatively close to each other.

Besides SA/V, surface topography may also affect the outcome of immersion studies, as shown in another study by Sinitsyna et al.^[^ ^20^ ^]^ The authors compared the dissolution behavior of S53P4 flame‐sprayed microspheres and melt‐derived, crushed and sieved granules of the same nominal size (45–90 µm). Ion release and surface layer formation were investigated under dynamic flow (0.2 mL min^−1^ for up to 24 h) of Tris‐HCl buffer solution, during which pH and released ion concentrations were measured constantly. Granules and microsphere composition differed only negligibly from the nominal one. Throughout the experiment, mass loss was significantly higher for granules than for microspheres; pH was higher for granules than for microspheres over the first eight hours only. The authors explain these effects with the larger SA/V ratio of the granules owing to a broader size distribution. In addition, there may be an effect of topography; the sharp corners and edges present on the granule surface result in a greater surface energy compared to that of the smooth microspheres, causing faster reactions with the solution to minimize that energy. Concentrations of silicon, calcium, and sodium ions in solution showed the same trend, while phosphate concentrations in solution showed the opposite trend, being lower for granules than for microspheres. Lower phosphate concentrations in solution during immersion experiments are typically related to faster apatite (or CaP) surface layer formation. In this study, however, the situation was more complex. Both granules and microspheres showed silica‐rich and mixed silica and CaP layers as early as four hours of contact with Tris‐HCl buffer solution. The silica‐rich layer was thicker for the granules than for the microspheres, caused by the faster ion release mentioned above. A CaP layer was detected on the microspheres at 24 h, while no pure CaP layer was detected for the granules. The authors explained this finding by the higher pH for granules at early time points. As shown in their previous study, discussed above, CaP layer formation is delayed or absent in high pH conditions, such as within a glass particle bed.^[^ ^12^ ^]^ In order to predict the dissolution kinetics of S53P4 microspheres during early stages, the authors used a shrinking core model based on the external mass transport coefficient, diffusion through the silica‐rich layer, and dissolution rate coefficient. Results suggest that this model is useful for predicting the dissolution mechanism of silicate‐based BG.

Porosity of BG implants is an important parameter for the ingrowth of cells in vivo, but it may also alter ion release and apatite surface layer formation, especially if the SA/V ratio changes with porosity. Aalto‐Setälä et al.^[^ ^21^ ^]^ prepared amorphous 3D porous scaffolds of S53P4 using optimized sintering parameters in order to avoid crystallization. In a dynamic dissolution experiment in SBF or Tris‐HCl buffer solutions, with a controlled flow rate of 0.2 mL min^−1^, these porous scaffolds were compared to freely packed granules. This study is unusual as completely amorphous porous S53P4 scaffolds were investigated, in contrast to many studies where sintered scaffolds are at least partially crystalline. This made it possible to compare granules and scaffolds without results being affected by the presence of crystalline phases. Granules in Tris‐HCl buffer solution showed much larger weight loss than the scaffolds (99 vs 73%). In SBF, this difference was less pronounced, as out of the two samples studied, one showed comparable weight loss (52% for granules vs 53% for scaffold), while a second sample in SBF only showed a weight loss of 38%. CaP layer formation was observed in SBF but not Tris‐HCl buffer solution, but the authors reported no differences in surface layer formation between granules and scaffolds. Relative ion concentration in solution, i.e., the percentage of ions (Si, Na, and Ca, with Na and Ca only being reported for Tris‐HCl buffer solution owing to the inherent concentrations of those two ions in SBF) dissolved from the glass, was higher for granules than for scaffolds, in agreement with the weight loss results. As these differences cannot be caused by the presence of (more chemically stable) crystalline phases, they likely originate from changes in relative surface area or in surface topography, i.e., the presence of sharp edges and corners on the granules as discussed above. However, as the authors did not characterize surfaces or surface area, this remains an assumption.

**Figure 3:**
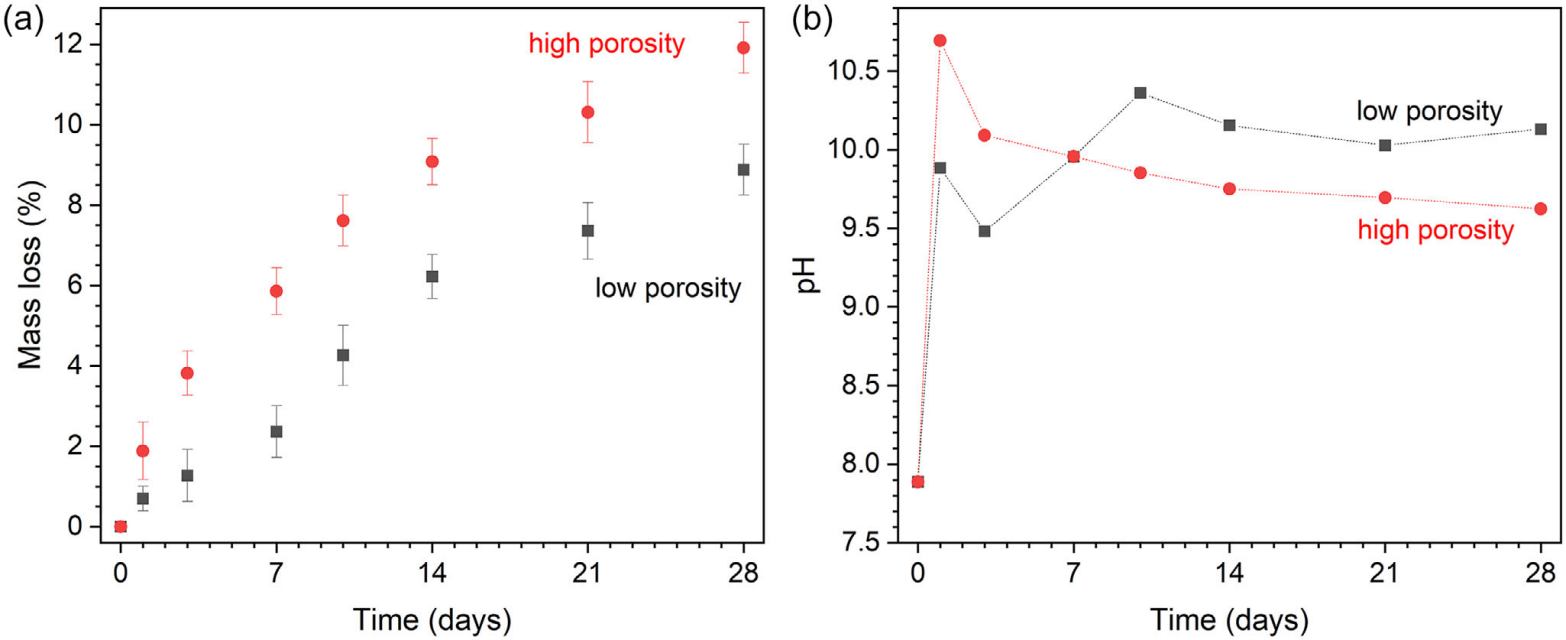
a) Mass loss and b) pH changes during immersion of S53P4 microspheres of low (black) and high porosity (red) in Milli‐Q water. Data for 45S5 are comparable. Error for pH data is less than the size of the symbols; lines are visual guides. Graphs plotted based on data presented by Islam et al.^[^ ^22^ ^]^

When the effect of porosity on the SA/V ratio of BG is characterized, the expected trend of faster reaction and degradation with increasing SA/V ratio, and, thus, porosity, becomes apparent. Islam et al.^[^ ^22^ ^]^ prepared porous microspheres of 45S5 and S53P4 by flame spheroidization to investigate the effect of porosity on ion release and surface layer formation during immersion. Microspheres were sieved to between 125 and 200 µm in size. While the authors refer to their samples as either “solid” or “porous”, all microspheres they prepared actually showed internal porosity of either 28.2 ± 3.5 µm (low porosity) or 67.9 ± 0.5 µm (high porosity), with the high‐porosity samples having been prepared by adding calcium carbonate as a porogen. Samples of high internal porosity showed a porous outer surface and highly interconnected internal porosity, while the samples of low internal porosity did not. Sample composition was close to the nominal one, with the exception of the outer surface of the high‐porosity samples, which was enriched in calcium and depleted in silicon and sodium compared to the bulk/nominal composition. During immersion in Milli‐Q water over 28 days, a mass loss of 12.6 and 11.9% was observed for high‐porosity samples of 45S5 and S53P4, respectively, while the low‐porosity samples only showed a weight loss of 8.9% each (**Figure** **3**a, data shown for S53P4 only). During immersion in Milli‐Q water, pH spiked for the high‐porosity samples (to over 11 for 45S5), then decreased slightly and remained relatively constant for the remaining time. pH for low‐porosity samples also increased rapidly during the first 24 h of the experiment, but did not reach as high a level, and pH kept increasing more slowly over the next days (Figure 3b, data shown for S53P4 only). Powder X‐ray diffraction patterns after immersion in SBF revealed that the high‐porosity samples showed apatite formation at 1 day of immersion, while the low‐porosity samples showed apatite diffraction peaks at three (45S5) or five (S53P4) days only. Taken together, the increased relative surface area of the high‐porosity samples caused much faster degradation compared to the low‐porosity ones.

Another interesting aspect of the study by Zhang et al.^[^ ^12^ ^]^ presented above is that local pH within a BG particle bed differs drastically from that one of the bulk solution (Figure 2b). Unlike other studies, the authors measured the solution pH within the particle bed as well as in the bulk solution, thereby giving a much better impression of pH conditions at or near the BG/solution interface. Results showed the final pH within the particle bed to be much higher than the final pH in the bulk solution (measured after removal of the glass particles); this difference is assumed to originate from poor diffusion among the particles. The higher pH within the particle bed shows that the pH of the bulk solution cannot be taken to correctly describe the conditions at the BG/solution interface, in agreement with real‐time Raman spectroscopy investigations of interfacial reactions of a borosilicate glass in contact with an aqueous solution showing a pH gradient of higher pH directly at the surface.^[^ ^23^ ^]^ Surface layer formation also depended on the position of the particles within the particle bed. Particles at the outer margin showed distinct reaction layers, while those inside the particle bed did not show any. These results confirmed previous findings by the same authors on immersed fiber bundles,^[^ ^24^ ^]^ where the outer fibers showed distinct and uniform layers, while fiber surfaces from within the bundle showed no reaction layers or very uneven ones only. This trend with location (outside vs within particle bed or fiber bundle) suggests that local conditions can vary greatly during immersion, influenced by diffusion and local supersaturation. It can be safely assumed that such factors also come into play when BG granules are implanted into the body, particularly at locations where fluid exchange is low, such as inside bone voids.

This effect of location (outside vs within particle bed or fiber bundle) can be explained by concentration effects. Increasing BG concentration during immersion studies has been shown to result in increased ion concentrations in solution but simultaneously reduced the rate of apatite precipitation, an effect which is likely to affect the outcomes of in vitro cell culture studies. Jones et al.^[^ ^25^ ^]^ investigated the influence of BG concentration in SBF using fine powders (< 5 µm) of melt‐derived 45S5; concentrations varied between 0.001 and 0.015 g mL^−1^. Solution pH increased with BG concentration, with pH at 22 h ranging from 7.4 to 8.7 from the smallest to the largest concentration; sodium concentrations in solution showed a similar increase with BG concentration. Concentrations of silicon in solution were lowest for the smallest BG concentration but showed no pronounced variation within the remaining BG concentrations. This is probably explained by the very limited silicon (or, more precisely, solubility of silicate species) solubility in solution, resulting in precipitation of silica gel as release increases.^[^ ^26^ ^]^ Calcium concentrations in solution increased up to BG contents of 0.005 g mL^−1^ but remained constant for larger BG concentrations. By contrast, phosphate concentration decreased as glass content in the solution increased. The latter may be related to precipitation of phosphates, either apatite or amorphous calcium phosphates (CaP), but this was not confirmed by either XRD, which showed very poor resolution, or FTIR spectroscopy, most likely because of the longest time period being too short for apatite to form and, thus, only amorphous CaP being present. The XRD pattern of the highest 45S5 concentration showed a diffraction peak corresponding to calcite, i.e., calcium carbonate. It has been shown that phosphate concentration in solution often is the limiting factor for precipitation of phosphate phases (both apatite and amorphous CaP), and increasing calcium concentrations in solution may therefore result in calcite precipitation.^[^ ^10^ ^]^

BG are known to crystallize easily during processing at high temperatures, and crystallization may be expected to reduce ion release and degradation, owing to crystalline materials typically being more chemically stable than amorphous ones of the same stoichiometry. Clupper et al.^[^ ^27^, ^28^ ^]^ compared the effect of sintering conditions (temperature and time) of tape‐cast and sintered 45S5 discs on ion release and surface layer formation during immersion in either SBF or Tris buffer solution. The authors prepared tape‐cast discs sintered for three hours at 800, 900, or 1000 °C or for six hours at 1000 °C. X‐ray diffraction showed the glass to have crystallized to a sodium calcium silicate, Na_2_Ca_2_Si_3_O_9_, with no differences with sintering conditions being apparent. Results of immersion experiments changed with sintering conditions, however, the Ca/P ratio, obtained from compositional analysis of the surfaces of the glass‐ceramics immersed in SBF, at the earliest time point of immersion in SBF (two hours) increased with sintering temperature or time (1.1 for the sample sintered at 900 °C, 1.45 for the sample sintered for three hours at 1000 °C and 2.2 for the sample sintered for six hours at 1000 °C). The authors also compared the ratio of FTIR bands for Si—O bending (near 450 cm^−1^) and P—O bending (near 602 or 575 cm^−1^). This Si/HA ratio also varied with sintering conditions, with the four samples falling into two groups for immersion times of 24 h or more, with samples treated at 1000 °C having much lower Si/HA ratios (between 0 and 0.5) than the samples treated at 800 or 900 °C (ratio between 1 and 2). After immersion in Tris buffer solution, differences in surface Ca/P ratio were less pronounced, with samples treated for three hours at 900 or 1000 °C giving similar results, and the one treated for six hours at 1000 °C giving lower values. The Si/HA ratio obtained from FTIR spectra after immersion in Tris buffer solution showed the three samples with the longest sintering time to be very similar; the ratio decreased from about 2.5 at one hour to close to 0 at 14 days. The sample sintered at 800 °C, by contrast, had much higher Si/HA ratios throughout, decreasing from about four at two hours to about 3 at 14 days. The authors interpreted these findings as originating from differences in the amount of remaining porosity after sintering and, subsequently, variation in relative surface area. For a more detailed interpretation, information on crystallinity, i.e., the relative amounts of crystal phase vs residual glassy matrix, or crystal morphology would be needed, but was unfortunately not provided by the authors.

Aalto‐Setälä et al.^[^ ^29^ ^]^ also studied sintered and crystallized samples of 45S5 and S53P4 during in vitro immersion, and they compared granules with sintered porous scaffolds and crushed sintered porous scaffolds. However, as they did not investigate phase composition, i.e., crystalline phases, after sintering in detail but only assumed the same crystal phases to be present as in either their own previous study on S53P4 or studies by other authors on 45S5, their results are difficult to interpret. Here, also, more detailed knowledge of crystallinity and crystal morphology would be helpful.

## 3 In Vitro Cell Culture Experiments

Despite some correlation between in vitro apatite mineralization and in vivo bone formation in simple glass systems,^[^ ^30^ ^]^ immersion experiments in acellular solutions cannot predict how the material tested will affect a living system.^[^ ^31^ ^]^ The testing solutions typically used are compositionally simple and do not contain proteins such as collagen, fibronectin, or vitronectin,^[^ ^32^ ^]^ which have been shown to affect apatite formation on BG^[^ ^33^ ^]^ and cell adhesion (e.g., by osteoblasts)^[^ ^32^ ^]^ on the apatite surface. In vitro cell tests, by contrast, are usually performed using culture media containing serum proteins in addition to physiological concentrations of inorganic ions. They are, therefore, despite limitations of their own, closer to real‐life conditions.

The inherent buffering system, as well as fluid flow and exchange in the living body, maintain the pH at relatively constant values, even after implantation of BG. By contrast, during in vitro studies, the ion exchange between BG and the surrounding aqueous solution results in a drastic pH rise, as experimental conditions are usually static (no fluid exchange) or semi‐static (fluid exchange at regular intervals only).^[^ ^18^ ^]^ For this reason, cell culture studies on BG often involve a pre‐conditioning step to control this inherent pH increase.^[^ ^34^ ^]^ While the exact pre‐conditioning parameters vary,^[^ ^34^ ^]^ they usually involve immersing the BG specimen in the cell culture medium for a defined period of time without adding any cells, to allow for the initial ion release and pH burst to occur without affecting cells. This pre‐conditioning also means that apatite (or calcium phosphate, CaP) surface layer precipitation often is induced before cells are added, which means that cells in vitro do not always get exposed to the BG surface but a CaP surface instead. In the body, by contrast, at very early stages after BG implantation, cells are exposed to the BG surface, although this surface is rapidly altered by protein adhesion and surface layer formation as mentioned above.

**Figure 4:**
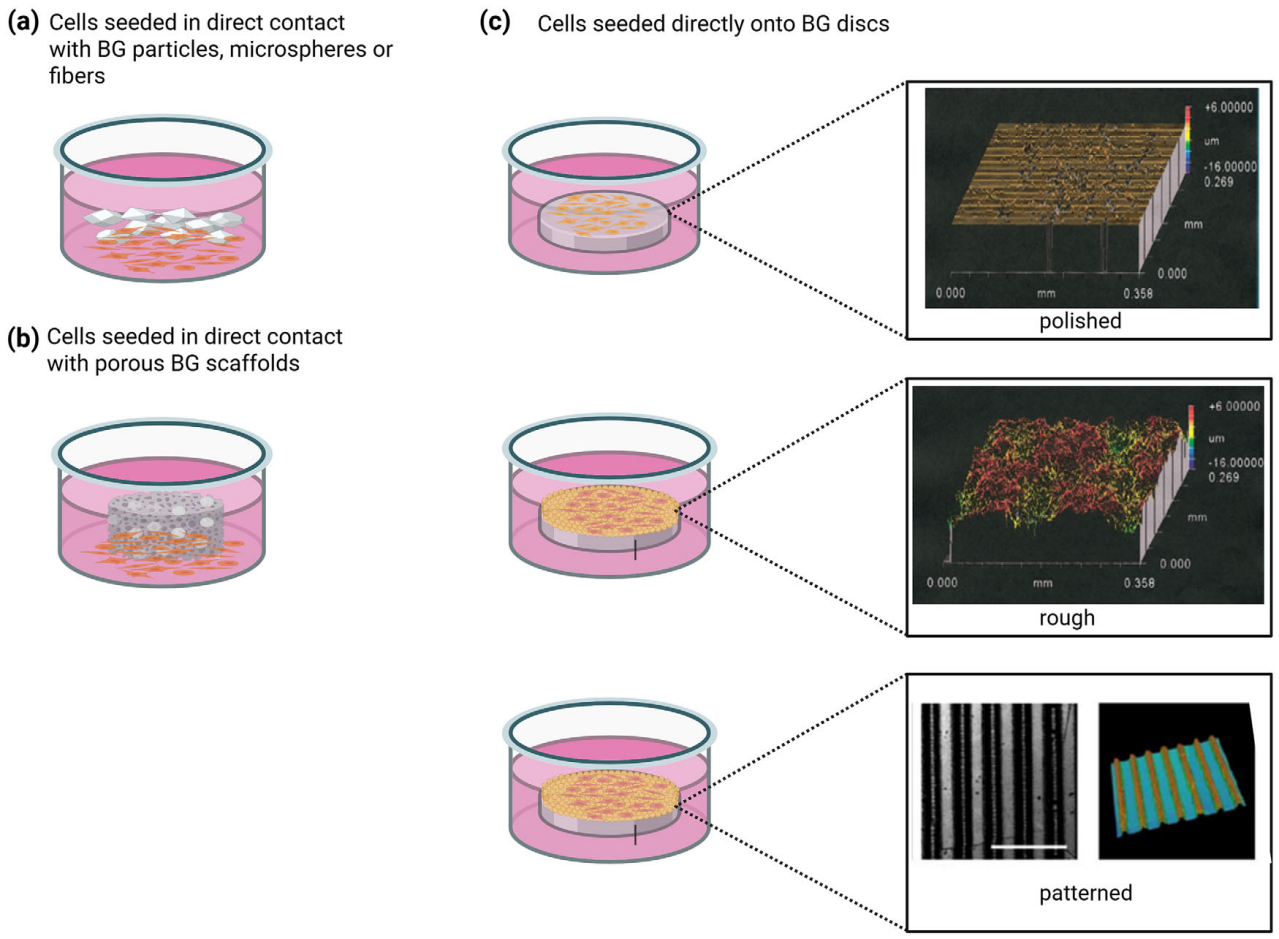
Different approaches for in vitro cell culture experiments, where cells are cultured in direct contact with a) BG particles, b) porous scaffolds, or c) on the surface of BG discs, which may differ in their surface structure or patterning (bottom scale bar = 100 µm). Figure created in BioRender. Horbert, V. (2025) https://BioRender.com/wue0cz7. Insets in (c) reproduced with permission.^[^ ^36^, ^37^ ^]^ Copyright 2004 & 2018, Wiley.

**Figure 5:**
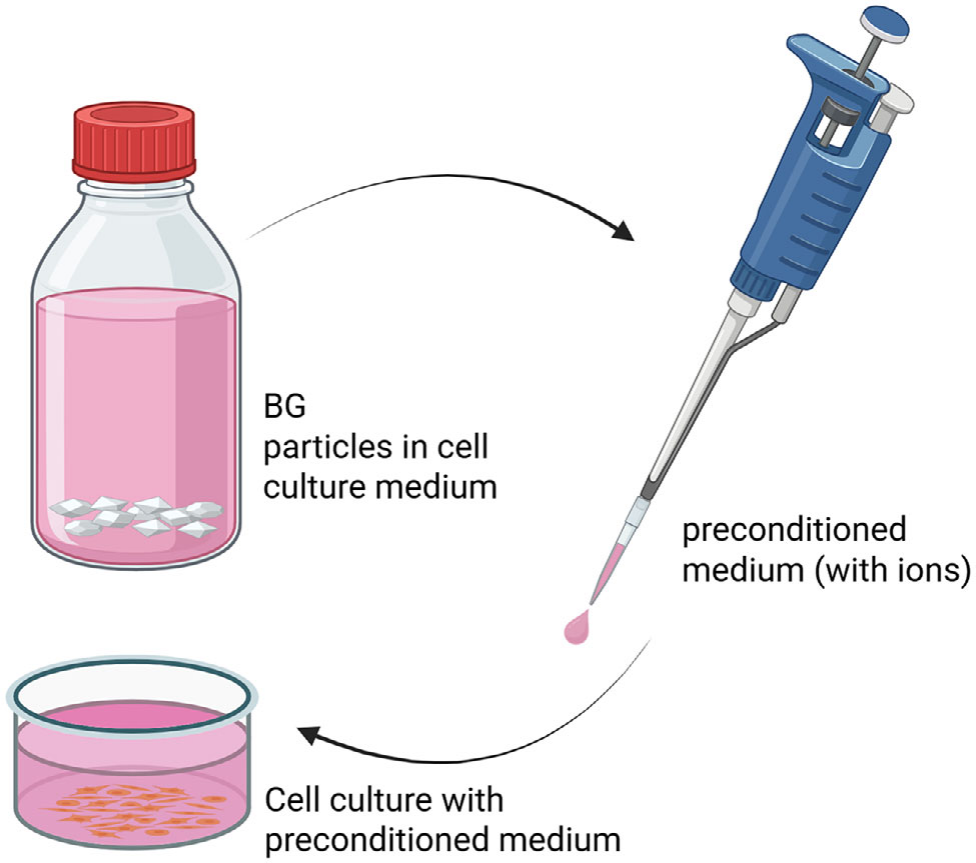
For indirect cell culture experiments, BG is immersed in the required cell culture medium under defined conditions (surface area to volume ratio, temperature, time). Afterward, the culture medium and BG are separated and only the medium, containing ions released from the glass, is used for the culturing of cells. Figure created in BioRender. Horbert, V. (2025) https://BioRender.com/wue0cz7.

When performing cell culture experiments, we distinguish between direct methods, where the cells are cultured in direct physical contact with the BG (**Figure** **4**), and indirect methods, where the BG may or may not be present in the culture medium but the cells are not in direct contact with it and are thus influenced indirectly via the ion release process the BG undergoes, such as changes in ionic concentrations or pH variations.^[^ ^35^ ^]^ A typical example of such an indirect method is the use of glass extracts (**Figure** **5**), where the BG is immersed in the culture medium under specific conditions for a defined period of time, but the medium is filtered (or in other ways separated from the BG) before being added to the cells. Experimental details of the studies included in this section are summarized in Table S2 (Supporting Information).

A series of experiments combined direct and indirect cell culture studies on 45S5, thereby providing clear evidence for the beneficial effect of ions released from BG had on cells. Xynos et al. investigated the effect of 45S5 on primary human osteoblasts by directly seeding the cells onto melt‐derived polished discs of 45S5 in vitro.^[^ ^38^ ^]^ The glass stimulated the growth and osteogenic differentiation of the cells, resulting in a more mature cell population capable of producing mineralized tissue within a relatively short time, compared to cells grown on a bioinert polymer control.^[^ ^38^ ^]^ When the same authors performed cell culture experiments using an indirect method, with cells being exposed to the ionic dissolution products of 45S5 rather than the actual material, results showed that these dissolution products, most likely soluble silica species,^[^ ^39^, ^40^ ^]^ helped to up‐regulate the expression of various genes, including a known inducer of osteoblast proliferation, in human primary osteoblasts.^[^ ^41^, ^42^ ^]^ Later experiments by Silver et al.^[^ ^43^ ^]^ showed that osteoblast ATP generation was enhanced by 45S5, independent of whether cells were in direct contact with the BG or exposed to its dissolution products only. These findings helped to understand that BG possess an inherent bioactivity besides apatite surface mineralization^[^ ^44^ ^]^ and that supplementation with growth factors is not needed for them, unlike for other bioceramics such as apatite in tissue engineering.

Further experiments using 45S5 dissolution products^[^ ^45^ ^]^ were intended to identify the optimum soluble silica concentration range for stimulating osteoblasts. While there were no significant differences between the two concentrations used (15 vs 20 µg mL^−1^), results showed that dissolution products were capable of creating an acellular environment supporting expression of osteoblastic phenotype, extracellular matrix deposition, and mineralization in vitro. These findings were further elaborated on by Jell et al.^[^ ^46^ ^]^ who showed that Raman spectroscopy is a useful tool to non‐invasively detect biochemical changes associated with the differentiation of fetal osteoblasts when cultured in the presence of dissolution products of 45S5.

Another study combining direct and indirect cell culture studies showed that ions released from BG can promote an anti‐inflammatory macrophage phenotype, Kajander et al.^[^ ^47^ ^]^ studied the effect of S53P4 on macrophages (murine‐derived RAW264.7 and J774‐Dual macrophage‐like cells), comparing direct (cells being cultured in direct contact with the glass granules, 500–800 µm) and indirect cell culture experiments (cells cultured using BG‐conditioned culture medium prepared by immersing 15 mg of S53P4 granules in 1 mL of culture medium for 48 h at 37 °C and 5% CO_2_). pH was measured in situ during cell culture using a fiber optic pH sensor or ex situ using a standard pH meter. Macrophages are part of the immune system, and they serve to fight and eliminate pathogens. To investigate macrophage function in response to exposure to S53P4 (either in particulate form or its dissolution products), the authors tested inflammatory interferon regulatory factor (IRF) and NF‐κB pathways in J774‐Dual cells. Results showed no difference between direct and indirect culture experiments. Nitric oxide produced by macrophages is an important signaling molecule in their immunometabolism, and this was investigated by measuring nitrite levels in RAW264.7 culture, corresponding to nitric oxide production. Again, no differences between direct and indirect experiments were observed. These findings suggest that S53P4 promotes an anti‐inflammatory macrophage phenotype, an effect not depending on direct contact between cells and BG, indicating that soluble BG components cause these changes. By contrast, macrophage RAW264.7 proliferation was slower in direct contact with S53P4 (and Al_2_O_3_ control) compared to cells cultured with conditioned medium, but no differences in macrophage morphology were observed. This effect may be related to mechanical disturbance, e.g., through movement of particles within the culture well, as it also appeared for the bioinert control of comparable particle size. Culture medium pH was about 0.2 units higher in contact with S53P4 compared to the control, while the difference was slightly less pronounced for BG‐conditioned medium. pH decreased with time, which the authors explained by macrophage metabolism, but it may also be related to dissolution of CO_2_ from the atmosphere (5% CO_2_) into the medium. But even with this pH decrease, culture medium containing or treated with S53P4 maintained the highest pH, highlighting the effect of BG dissolution on the culture medium.

BG are known to increase the solution pH during immersion, with the maximum pH depending on BG concentration and SA/V ratio. Silver et al.^[^ ^43^ ^]^ therefore investigated the effect of culture medium pH on osteoblasts. Culture medium was supplemented with 50mM Tris buffer solution of either pH 7.4 (i.e., of the same pH as the original culture medium) or pH 8, thus preparing culture medium having an initial pH of either 7.4 or 7.8. The presence of 45S5 granules caused further alkalinization of each medium. Results showed a positive correlation between culture medium alkalinity and lactate production and, hence, ATP generation. Cell metabolic activity, quantified by MTT staining, was not affected. The authors further showed that 45S5 increased not only pH and ion concentrations extracellularly but also intracellularly, and they interpreted their results as an indication for alkalization, possibly being the major component of the beneficial effect 45S5 had on bone growth. They further pointed out that in vivo, with extracellular space being small and extracellular fluid movement potentially being slow, the presence of 45S5 or similar BG could potentially lead to pronounced changes in tissue pH.

Another study aimed at elucidating the effect of BG concentration on cells in vitro, showing that the pH increase caused by the BG particles is not necessarily detrimental to cells. Sarin et al.^[^ ^48^ ^]^ investigated the inhibitory effects of various concentrations of S53P4 on HaCaT cell viability, proliferation, and inflammatory response in two types of culture medium (Dulbecco's Modified Eagle's Medium, DMEM, and Keratinocyte Growth Medium, KGM). S53P4 granules (0.5 to 0.8 mm particle size range) were stored in a rotating mixer in either medium at concentrations between 0.5 and 10% for five days at 4 °C. Afterwards, the medium was transferred to a 37 °C incubator for 24 h before use. pH of the medium (at 21 °C) was measured before use. Unfortunately, the authors did not state whether the remaining BG granules were removed from the culture medium before use. HaCaT cells (immortalized human epidermal keratinocyte‐derived cell line) were incubated for two days in contact with BG‐treated culture medium. Afterwards, cell viability was determined using the MTT assay or cell counting. Despite culture medium pH increasing significantly with increasing BG concentration (from 7.74 for untreated DMEM to 8.51 for DMEM with 10% BG and from 7.63 for untreated KGM to 8.61 at 10% BG), results from cell culture testing were non‐conclusive. Culture experiments were performed in three independent replicates, and concentration effects varied between the groups. This was particularly noticeable in DMEM, where one group showed a statistically significant decrease in viability with increasing BG concentration, another showed the reverse, while the third group showed no statistically significant differences. In KGM, two out of three groups showed a statistically significant decrease in viability, while the third group did not. pH changes are usually considered detrimental for cells, but these results show that the actual cell response does vary. In this particular study, the reason may be the relatively moderate pH increase, which seems to have been tolerated by at least some of the cell groups in the study.

Sarin et al.^[^ ^48^ ^]^ further investigated the effect of BG concentration by performing scratch assays in direct contact with S53P4 granules. Confluent monolayers of HaCaT cells were scratched to create an ≈0.5 mm wide scratch wound. Afterwards, BG granules were added at various concentrations (0.9, 1.7, or 3.5%); the control group received no BG. Confluence in the scratch area was investigated at 24 and 48 h. The control group showed confluence levels of 0 to 30% (with a standard deviation, SD, of 12.6) at 24 h and 10 to 100% (SD 34) at 48 h. At 0.9% BG, confluence in the scratch area was between 0 and 20% (SD 8.2) at 24 h and between 0 and 50% (SD 20) at 48 h. At 1.7% BG, confluence levels were even lower, being between 0 and 10% at either time point (SD 4.1 and 4.9, respectively), while the highest BG concentration (3.5%) showed no cell growth in the scratch area at either 24 or 48 h. Instead, cell debris and widening of the scratch area were observed. Unlike the cell viability results, these scratch tests show a detrimental effect of BG, particularly at higher concentrations. This was confirmed by light microscopy of confluent HaCaT layers, where undisturbed monolayers were observed at a distance from BG particles but dead cells in the immediate BG vicinity. Decreased concentrations of pro‐inflammatory cytokines interleukin‐6 and interleukin‐8 in BG‐containing culture media suggest that cells near the glass granules underwent apoptosis (controlled cell death) rather than necrosis. Whether these results originate from pH effects or are caused by physical contact between cells and BG granules remains, however, unclear.

While these results show that BG concentration is likely to influence experimental outcomes of cell culture tests, the actual effects may depend on the particle size used, as shown in a study investigating the influence of both size and concentration, as discussed in the following section.^[^ ^49^ ^]^

Despite the known beneficial effects of ions released from BG, the rapid pH increase upon contact between 45S5 and cell culture medium potentially represents a concern during early stages of cell culture, depending on the type of cell used. Pre‐conditioning of BG is a well‐known approach used before in vitro cell culture experiments, in an attempt to make up for the lack of fluid exchange present in vivo but not in vitro. For this reason, Hattar et al.^[^ ^50^ ^]^ investigated the effect of pre‐conditioning of 45S5 granules on MG63 osteoblast‐like cells by comparing cells cultured in the presence of 45S5 granules either pre‐conditioned in 0.2 M Tris buffer solution of pH 7.25 for 48 h or non‐preconditioned. RT‐PCR results showed that cells expressed all studied markers under either condition. Although some differences appeared, the authors concluded that BG pre‐conditioning did not affect osteoblast mRNA expression.

## 4 Effects of BG Morphology on Cells Cultured In Vitro

**Figure 6:**
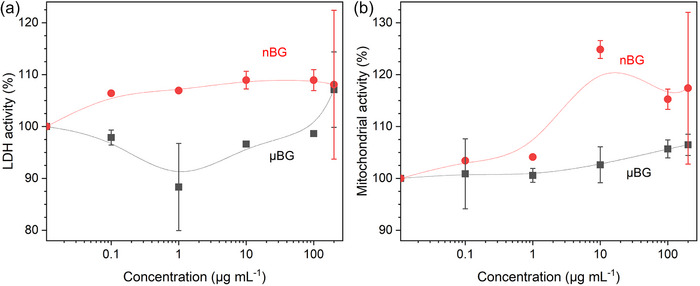
a) LDH activity and b) mitochondrial activity of MG63 cells cultured on µBG or nBG of various concentrations. Differences were statistically significant for concentrations of a) 0.1, 1, 10, 100 µg mL^−1^ and b) 10 and 100 µg mL^−1^. (Data normalized to 0 µg mL^−1^ = 100%.) Graphs plotted based on data presented by Mačković et al.^[^ ^13^ ^]^

The changes in ion release with SA/V ratio during immersion of BG in aqueous solutions are reflected in the outcomes of cell culture experiments as well. Mačković et al.^[^ ^13^ ^]^ evaluated the in vitro cytocompatibility of 45S5 in two particle size ranges, flame‐sprayed nanoscale particles (20–60 nm; nBG) and melt‐derived microparticles (D_50_, 10 µm; µBG), using MG63 osteoblast cells. They included glass particles across a concentration range of 0.1 to 200 µg mL^−1^ over a 48‐h period. The study assessed cell viability, number, morphology, and specific alkaline phosphatase (ALP) activity. Results indicated no cytotoxic effects for either nBG or µBG, even at the highest concentration of 200 µg mL^−1^. The authors reported that even at a relatively high concentration of 1000 µg mL^−1^ of nBG, 81% cell viability was retained (the corresponding data were not shown). Notably, nBG at concentrations of 10 and 100 µg mL^−1^ significantly enhanced cell viability compared to µBG, as indicated by mitochondrial activity exceeding 100% relative to control. Similarly, a greater number of cells were observed by light microscopy on nBG surfaces within the concentration range of 0.1 to 100 µg mL^−1^. The morphology of MG63 cells remained largely unaffected by both particle types, retaining their typical elongated and polygonal phenotype, even though nBG exhibited aggregation at the highest concentration. Despite this early‐stage aggregation, nBG induced higher MG63 cell activity, as evidenced by increased specific ALP activity at 48 h of incubation compared to µBG. This enhanced activity may be attributed to the dissolution products of the BG, which are known to stimulate the expression of several osteoblastic genes,^[^ ^51^ ^]^ particularly those associated with ALP activity, primarily driven by the release of silicon and phosphorus. LDH activity, a measure of the number of cells attached to the BG, was also higher for nBG than for µBG for most of the concentrations studied, while mitochondrial activity was higher for nBG at higher concentrations (**Figure** **6**).

The reduction in ion release with increasing particle size or decreasing SA/V ratio may also lead to a reduction in vascular endothelial growth factor (VEGF) expression, as shown by Detsch et al.^[^ ^49^ ^]^ The authors investigated the influence of S53P4 particle size and concentrations on fibroblast cell (CCD‐18CO) response and VEGF release for up to 72 h in vitro. Three BG size ranges (0.5 to 0.8, 1.0 to 2.0, and 2.0 to 3.0 mm) and concentrations (0.01, 0.1, and 1% wt/vol) were included. Observations revealed no alterations in cell morphology across the analyzed size ranges and concentrations, indicating good biocompatibility. Cell proliferation and viability also did not show any trends with either particle size range or concentration. By contrast, trends in VEGF expression did vary with particle size; the smallest granules caused increased VEGF expression with BG concentration, while the largest granules exhibited an inhibitory effect on VEGF release with increasing BG concentration. Results showed no clear trend for the intermediate size range. The authors hypothesized that the inhibition of VEGF for the largest particle sizes may be attributable to its small SA/V ratio, exerting an influence on the dissolution behavior of the glass. This finding of particle size affecting VEGF release may be an important basis for the targeted optimization of BG.

**Figure 7:**
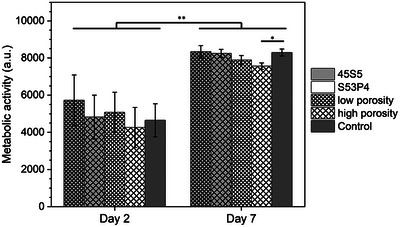
Metabolic activity of 3T3 cells exposed to 45S5 or S53P4 low or high porosity microsphere ion extracts at day 2 and day 7 of indirect culture (* *p* < 0.05; ** *p* < 0.0001). Graph plotted based on data presented by Islam et al.^[^ ^22^ ^]^

While variation in porosity‐induced SA/V ratio has been shown to directly influence ion release, this may not necessarily affect the outcome of in vitro cell culture studies. Islam et al.^[^ ^22^ ^]^ performed cytocompatibility studies on their porous flame‐sprayed microspheres, described in the section on acellular immersion experiments above. They performed indirect studies by treating 3T3 mouse fibroblasts with DMEM containing BG dissolution products and direct experiments. While the results of acellular immersion showed pronounced differences with porosity, no differences were observed in cell metabolic activity at two or seven days between high‐ and low porosity samples in indirect cell culture studies. Only high‐porosity S53P4 at seven days showed a statistically significantly lower metabolic activity compared to the control, possibly owing to high solubility (**Figure** **7**). Imaging after both indirect and direct cell culture experiments confirmed these findings.

While the BG‐induced pH increase is often described as at least potentially negative, it may improve cell adhesion during conditions associated with low pH such as hypoxia. Pérez‐Tanoira et al.^[^ ^52^ ^]^ performed cell culture experiments using human osteoblast‐like SaOS‐2 cells on polished S53P4 discs in either the presence or absence of S53P4 granules. Experiments were performed either in normal atmospheric conditions (20.9% O_2_; 0.35% CO_2_) or in hypoxia (6% O_2_; 7% CO_2_), i.e., conditions simulating those in bone cavities or in chronically infected bone. As described above, BG granules typically increase the pH of the surrounding medium, and this pH increase was observed here as well. In normoxic conditions, the presence of BG granules and the associated pH increase did not affect cell adhesion and cytoskeletal organization. Hypoxic conditions, however, are associated with lower pH, which typically impairs cell adhesion. Here, the addition of BG granules moved the pH closer to the normoxic one, and results suggest that this BG‐caused pH increase enhanced cell adhesion under hypoxic conditions by neutralizing local pH. The authors concluded that owing to improved cell adhesion in hypoxic conditions, S53P4 granules have potential as a good filling material for infected cavities where such low pH conditions prevail, and, indeed, S53P4 granules are successfully used for the treatment of chronic bone infections.^[^ ^53^ ^]^

Results of immersion experiments on partially crystallized samples have shown that BG crystallization may present a challenge for the interpretation of experimental outcomes, as discussed above. Once moving to cell culture experiments, results become even more difficult to interpret, especially if no in‐depth characterization of crystal phases and the amount of crystallinity is provided. Gough et al.^[^ ^54^ ^]^ studied the response of human primary osteoblast cells to tape‐cast discs of 45S5, sintered at for three hours at 800, 900, or 1000 °C or for six hours at 1000 °C. Preparation of the materials has been described by Clupper at al.,^[^ ^27^, ^55^ ^]^ who also characterized the materials, showing that during sintering, samples had crystallized to a sodium calcium silicate as described above. Silicon and sodium release from the samples and pH increase of the culture medium during immersion decreased with sintering temperature and time, similar to what was detected in Tris buffer solution.^[^ ^27^ ^]^ Apatite surface layer formation in DMEM was fastest for the sample sintered for six hours at 1000 °C, which is in contrast to the finding in SBF or Tris buffer solution.^[^ ^27^, ^28^ ^]^ At 90 min, very few cells attached to the 800 °C sample, while significantly more cells attached to the other samples. At 24 h, still hardly any cells attached to the 800 °C sample, samples sintered for three hours at either 900 or 1000 °C showed the largest number of attached cells, while the one sintered at 1000 °C for six hours showed intermediate numbers. Samples sintered at 900 °C and 1000 °C (six hours) showed the largest occurrence of apoptosis, while the sample sintered at 800 °C caused mainly necrosis. Bone nodule formation was observed mainly on the sample sintered at 1000 °C for three hours, and significantly lower nodule numbers were found on the 900 °C sample. The other two samples showed no formation of nodules. The authors concluded that an intermediate level of ion release or surface layer formation may be ideal for cells. However, in our view, this study highlights the fact that results from immersion experiments can only to some extent help to predict the results of in vitro cell culture tests. Results of cell culture experiments on tape‐cast sintered discs agree with the acellular immersion results of these samples (by Clupper et al.,^[^ ^27^, ^28^ ^]^ see above) insofar as results showed pronounced variation with sintering conditions despite the crystal phase of all samples being the same. This further highlights the need for more detailed materials analyses when working with crystallized glasses.

## 5 Effects of BG Surface Features on Cells Cultured In Vitro

**Figure 8:**
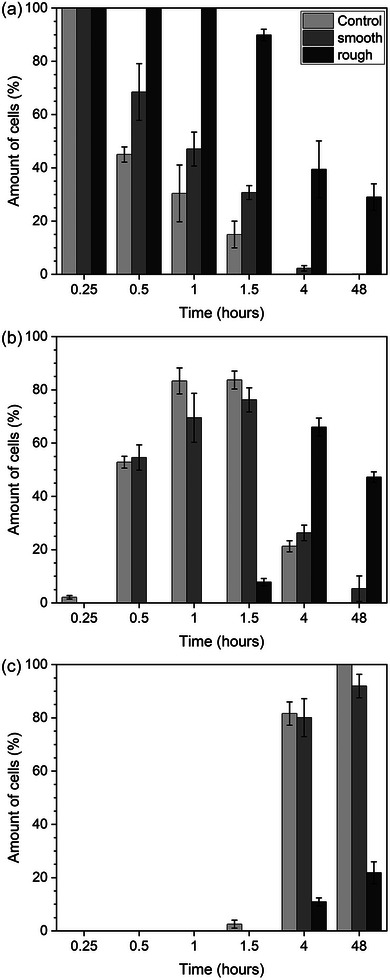
Relative amounts of cells showing a a) poor, b) intermediate, or c) good level of actin organization on smooth or rough 45S5 surfaces or on controls over time from 15 min to 48 h. Graphs plotted based on data presented by Gough et al.^[^ ^36^ ^]^

When culturing cells on BG monoliths, such as discs, the topography of the surface, e.g., whether it is polished or not, has a pronounced effect on cell behavior and properties. Gough et al.^[^ ^36^ ^]^ studied the effect of surface roughness of 45S5 discs (the two upper insets in Figure 4) on attachment, spreading, and mineralized nodule formation of primary human osteoblasts. Discs were either polished to a 3 µm finish (peak to valley distance, PV, 4.4 µm; arithmetical mean deviation of the assessed profile, R_a_, 0.045 µm) or had a rough surface (PV 21.3 µm; R_a_ 2.027 µm). Cell attachment and spreading were evaluated using actin cytoskeletal staining and cell scoring. Cell morphology differed between smooth and rough surfaces, with those on smooth surfaces appearing more spread and flattened, while those on rough surfaces had a more rounded, spiky appearance. This suggests that cells growing on the rough surface did not reach an ideal level of actin organization within 48 h. The authors therefore used FITC‐conjugated phalloidin staining to visualize actin organization in the cells grown on smooth or rough 45S5 surfaces or on controls (Thermanox). Cells could be organized into three groups: those with a poor level of actin filament organization, those with some level of radially oriented filaments, and cells showing well‐formed actin filaments parallel to the long axis of the cell. For each of the three materials, the level of actin organization increased over time (**Figure** **8**). Still, results showed pronounced differences between control, smooth, and rough 45S5 surfaces. Actin filament organization was much better on smooth than on rough 45S5 surfaces, nearly reaching that observed on the control. Poor levels of actin organization did not seem to negatively affect bone nodule formation on rough surfaces, however. Nodules formed on both smooth and rough surfaces, but numbers were significantly higher on the rough surfaces, possibly enabling accelerated bone formation in vivo. The authors explained this discrepancy with the involvement of cell filopodia (“microspikes”) in substrate surface sensing and rough surfaces allowing for a larger contact area between cell and substrate, even if the cell is more rounded. However, as apatite precipitation was faster on rough surfaces, this may also have affected bone nodule formation.

The effects of surface topography on cells can become even more pronounced if the BG surface shows a distinct patterning. Pföss et al.^[^ ^56^ ^]^ created micrometer‐scaled groove and ridge patterns on the surface of 45S5 by a direct casting method, resulting in a micro‐structured surface free of defects. Surface patterns consisted of ridges with a 10 µm width, grooves with a 30 µm width, and groove depths of either 8 (str8) or 15 µm (str15); polished surfaces were used as controls. Compared to cells on polished BG samples, cells on structured surfaces spread and formed long directional extensions. As a result, the cellular morphology appeared oriented along the direction of the grooves, suggesting that micro‐structuring of BG surfaces may cause cellular contact guidance. Höner et al.^[^ ^37^ ^]^ performed cell culture experiments on these structured BG surfaces using human mesenchymal stem cells (hMSCs), which have the potential to differentiate into osteoblasts (bone‐forming cells), and murine RAW264.7 progenitor cells, which can differentiate into osteoclasts (i.e., bone‐resorbing cells). Cells adhered to the surfaces, and on the structured surfaces, hMSCs exhibited a more elongated shape than on polished ones, with the cell body often remaining in the groove between the two ridges. Cells were mainly oriented parallel to the structure, with about 61% of cells oriented within 10° of the groove direction on str8 and over 88% on str15. However, staining showed that fewer focal adhesion points were formed on structured surfaces than on polished ones. Interestingly, structured surfaces induced effects beyond cell orientation. Generally, str15 showed higher protein levels and osteogenic marker gene expression than the other surfaces; TRAP gene expression of RAW264.7 cells increased nearly nine‐fold on structured 45S5 compared to polished 45S5 surfaces, suggesting the differentiation into osteoclast‐like cells.

**Figure 9:**
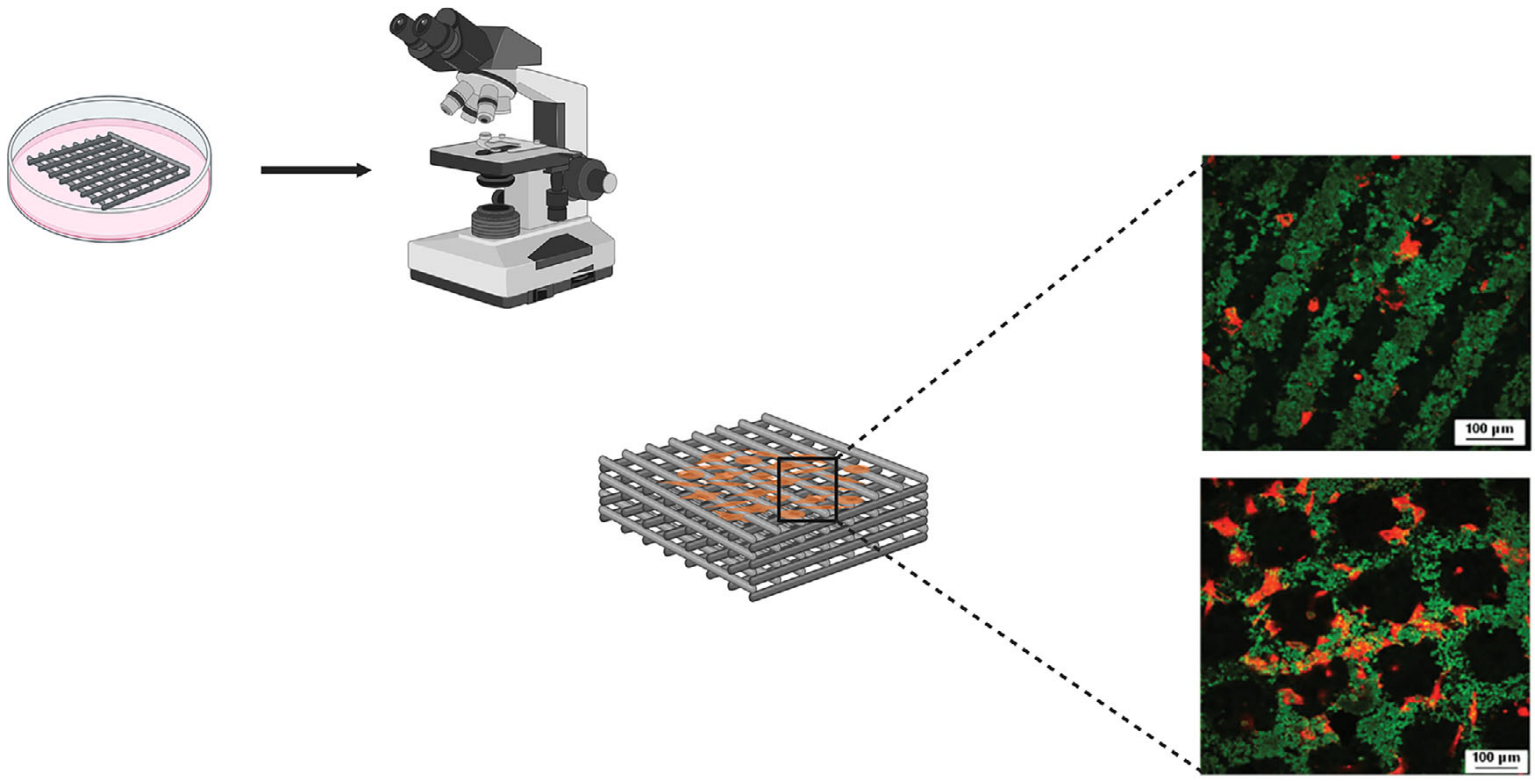
BG surface structure or patterning may influence the outcome of cell culture or even in vivo experiments. Studies have shown variation in surface features to affect cell adhesion, alignment, or spreading (cells presented stained in red, with the BG surface presented in green). Figure created in BioRender. Horbert, V. (2025) https://BioRender.com/wue0cz7. Micrographs reproduced with permission.^[^ ^57^ ^]^ Copyright 2012, Wiley.

Detsch et al.^[^ ^57^ ^]^ also investigated micro‐structured surfaces of 45S5 and compared them to flat 45S5 surfaces. Here, a soft lithography technique was used to introduce micro‐structured surface patterns in a sintered BG substrate.^[^ ^5^ ^]^ Therefore, both structured and flat surfaces possessed an inherent roughness owing to the sintered nature of the substrate, which is in contrast to the defect‐free patterned surfaces used in the studies by Pföss et al.^[^ ^56^ ^]^ and Höner et al.^[^ ^37^ ^]^ Two surface patterns were studied, equally spaced squares with a side length of 100 µm and a separation of 100 µm, and parallel ridges separated by 50 µm wide grooves. Flat, non‐patterned surfaces served as control. Owing to the poor sintering properties and pronounced crystallization tendency of 45S5,^[^ ^5^, ^58^ ^]^ the authors assumed their samples to be partially crystalline but did not analyze for crystalline phases. MG63 osteoblast‐like cells and rat mesenchymal stem cells (rMSC) were used in cell culture experiments. MG63 showed an elongated morphology, characterized by a larger aspect ratio, which on patterned surfaces aligned along the edges of the squares and grooves, while rMSC morphology was more evenly spread out on all surfaces, independent of patterns (**Figure** **9**). As a consequence, MG63 seemed to experience more contact guidance than rMSC; however, owing to the inherent sintering roughness, results were not as clear as in the study by Höner et al.^[^ ^37^ ^]^

Cell adhesion can further be controlled by surface functionalization. Azizi et al.^[^ ^59^ ^]^ used surface silanization and fibronectin coating of polished S53P4 discs to study cellular adhesion of mouse embryonic fibroblasts. BG discs were either surface‐silanized, surface‐silanized followed by fibronectin‐coating, or untreated. Time‐lapse imaging of cells showed that basic surface silanization significantly decreased cell movement on the glass surface, indicating that strong adhesion was achieved. By comparison, fibronectin grafting had a negligible effect on cell movement. Cell proliferation was not impacted by either treatment. Immunostaining of focal adhesion points revealed that untreated S53P4 resulted in considerably lower cell‐substrate interactions compared to the two treated surfaces, resulting in reduced cell adhesion on untreated surfaces. Cells on silanized surfaces appeared elongated, whereas cells on fibronectin‐grafted surfaces showed a more symmetrical appearance. Additionally, fibronectin‐grafted surfaces showed larger adhesion sites. As the culture medium contained serum proteins, including fibronectin, the biological activity of untreated or silanized surfaces was probably enhanced and, thus, more similar to fibronectin‐treated surfaces.

## 6 BG Morphology Effects on In Vitro Antimicrobial Activity

We have recently reviewed the antimicrobial effects of BG more generally^[^ ^60^ ^]^ and include here only the small number of studies directly comparing different BG morphologies (Table S3, Supporting Information).

The increase in BG degradation with increasing SA/V ratio improves BG antimicrobial properties, mostly through an increase in osmotic pressure and pH rise. Zhou et al.^[^ ^61^ ^]^ studied the antibacterial and antibiofilm effect of S53P4 and 45S5 of varying particle size (32–125 µm, 90–710 µm, 500–710 µm, or 1‐2 mm). Four bacterial strains, S*treptococcus gordonii*, *Veillonella parvula*, *Pseudomonas aeruginosa*, and Methicillin‐resistant *Staphylococcus aureus* (MRSA), were selected, and incubation periods were 24, 48, or 72 h. The overall trend in bacterial inhibition was largely influenced by the BG particle size, smaller particles exhibited a more pronounced antibacterial effect, significantly restricting the growth of the tested bacterial strains. The inhibitory effect on certain bacteria (V. parvula and P. aeruginosa), on the other hand, was not substantially different from that of the untreated controls after 48 h of treatment with larger particle sizes of S53P4. This phenomenon was attributed to enhanced BG dissolution for larger SA/V ratios, causing environmental changes such as variations in osmotic pressure and pH for the smaller particles but less so for the larger ones. A trend similar to that observed for antibacterial effects was also evident for antibiofilm activity, with smaller particles demonstrating significantly greater biofilm reduction compared to larger ones.^[^ ^61^ ^]^ The authors attributed this to the increased surface interaction of smaller particles with bacteria, which enhances their ability to disrupt and degrade biofilms. The study also indicated that, compared to S53P4, 45S5 particles with smaller size ranges exhibited greater effectiveness against the selected bacterial strains and their biofilms.

**Figure 10:**
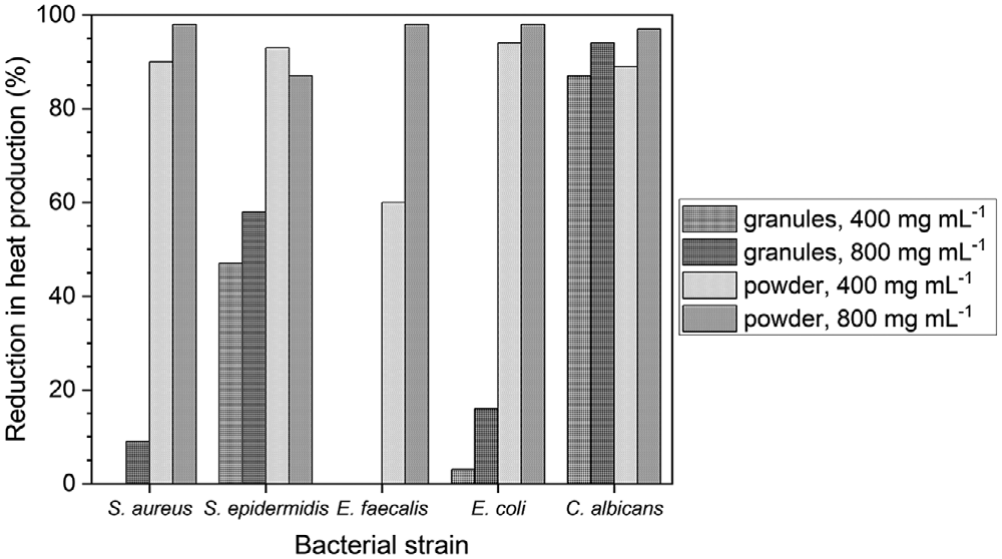
Heat production J) a measure of bacterial metabolic activity of various bacterial strains grown in the presence of S53P4 granules (500–800 µm) or powder (< 45 µm) at two concentrations (400 or 800 mg mL^−1^) for 24 h. Results are presented as relative reduction (%) compared to bacteria grown in the absence of S53P4. Graph plotted from data presented by Gonzalez Moreno et al.^[^ ^62^ ^]^

Isothermal microcalorimetry (IMC) is a highly sensitive technique allowing real‐time monitoring of microbial metabolism. Gonzalez Moreno et al.^[^ ^62^ ^]^ used this method to evaluate the antimicrobial effects of S53P4 particles of two size ranges (500–800 or < 45 µm) and concentrations (400 or 800 mg mL^−1^). Results were compared to those obtained using the standard method of colony‐forming unit (CFU) counting. Overall, heat production, a measure of bacterial metabolic activity, was reduced more by BG in the powder form (< 45 µm) and by higher concentrations (**Figure** **10**). Regardless of BG size or concentration, the metabolic activity of *Candida albicans* was reduced significantly, with reductions between 87 and 97% at 24 h. Granules at either concentration only caused minor reductions in heat production of *Staphylococcus aureus, Enterococcus faecalis*, and *Escherichia coli*, which for *E. faecalis* the authors explained by its higher tolerance to alkaline environments. Metabolic activity of *Staphylococcus epidermidis* was reduced by 47 and 58% for granules at low and high concentration, respectively. Antimicrobial activity of BG is mainly determined by their release of alkali metal cations and the resulting pH increase of the culture medium,^[^ ^63^ ^]^ with both effects being more pronounced at larger SA/V ratio, i.e., smaller particle size, and higher concentration.

Surface roughening is a known approach for reducing bacterial adhesion on BG.^[^ ^60^ ^]^ For BG, surface structuring by femtosecond laser can result in a combination of physical (i.e., via topography) and chemical antimicrobial action. Shaikh et al.^[^ ^64^ ^]^ created surface structuring on polished 45S5 discs using femtosecond laser modification. Depending on the scanning speed, roughness, characterized as average profile height (*R_a_*), quadratic mean of profile height (*R_q_*), and maximum profile height (*R_t_*), can be adjusted. Only the maximum surface roughness (*R_a_* = 6.3 µm, *R_q_* = 7.7 µm, and *R_t_* = 43.7 µm) prevented bacterial adhesion. In addition to inducing roughness, surface treatment also resulted in the formation of crystalline phases on the surface, including combeite, calcium hydroxide, and calcium carbonate. The authors interpreted this presence of calcium hydroxide, together with a more pronounced pH increase caused by the sample with maximum roughness, as contributing a chemically induced antimicrobial activity in addition to its physically induced one.

**Figure 11:**
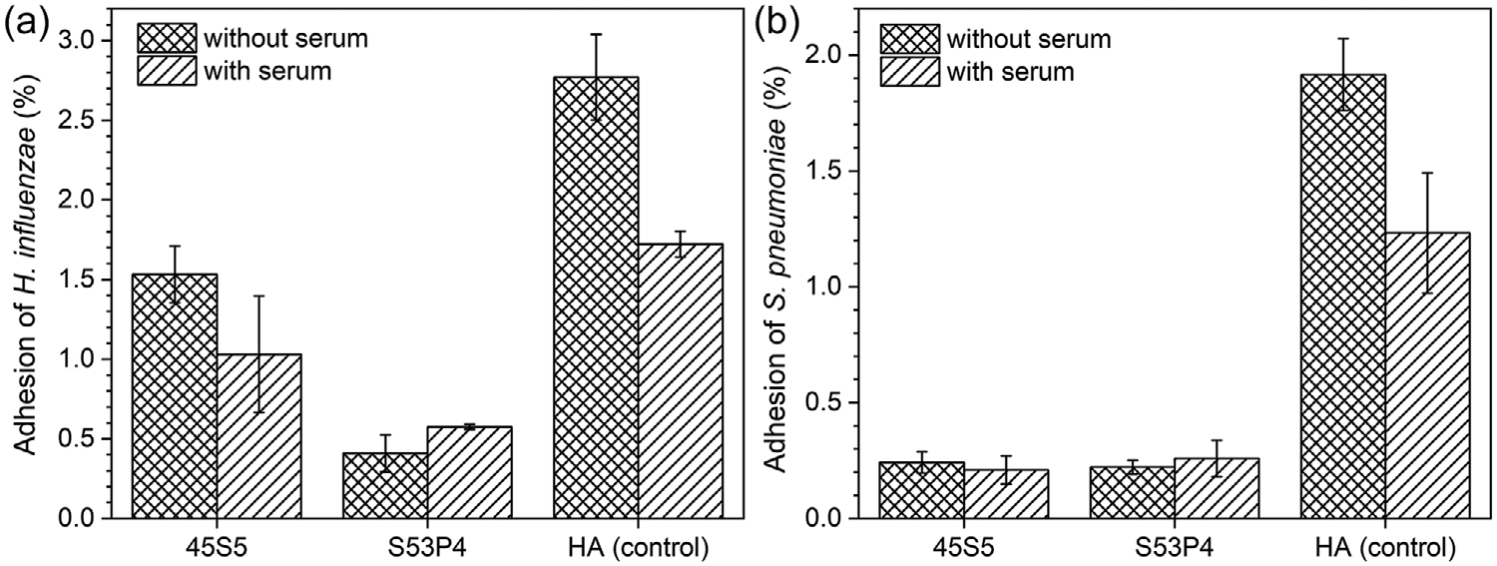
Percentage (mean ± range; n = 3) of bacteria, a) *H. influenzae* and b) *S. pneumoniae*, adsorbed to granules of each material either with or without serum pre‐treatment. Graphs plotted from data presented by Stoor et al.^[^ ^65^ ^]^

The inherent antimicrobial properties of BG mean that pre‐treatment, which improves the antimicrobial activity of other materials such as hydroxyapatite, does not result in further improvement of the antimicrobial activity of BG. Stoor et al.^[^ ^65^ ^]^ investigated the efficacy of S53P4 and 45S5 against two pathogens commonly associated with respiratory tract infections, *Haemophilus influenzae* and *Streptococcus pneumoniae*, in order to evaluate both BG for their suitability as interpositional graft materials in the surgical repair of nasal septal perforations. Adhesion tests were performed using granules (315–500 µm) of each material with or without human serum pre‐conditioning. Serum pre‐treatment seemed to reduce adhesion of either strain to the hydroxyapatite control, although the authors do not mention statistical significance (**Figure** **11**). By contrast, no pronounced effect of serum pre‐treatment was noticeable for the two BG.

## 7 Morphology Effects In Vivo, Animal Experiments and Clinical Studies

A recent paper reviewed 48 publications documenting clinical trials on BG for various indications.^[^ ^66^ ^]^ Overall, more than 700 patients were included in prospective and retrospective studies and case series. Bioglass 45S5, also known under trade names such as PerioGlas or BioGran, has been investigated in more than 20 clinical studies since 1997, primarily in the fields of periodontal therapy, bone augmentation, and dental implantology.^[^ ^67^, ^68^, ^69^, ^70^, ^71^, ^72^, ^73^, ^74^, ^75^, ^76^, ^77^, ^78^, ^79^, ^80^ ^]^ BonAlive S53P4 is primarily used in orthopedic surgery, for the treatment of chronic osteomyelitis, and in reconstructive craniofacial surgery. Since 2000, more than 25 clinical studies have been published.^[^ ^53^, ^66^, ^81^, ^82^, ^83^, ^84^, ^85^, ^86^, ^87^, ^88^, ^89^ ^]^ While in vivo studies mostly focus on BG granules, a small number of publications allow us to get insight into how BG morphology affects the outcomes of either animal experiments or clinical studies (Table S4, Supporting Information). Owing to the small number of in vivo studies focusing on comparing different BG morphologies, we also included studies on compositions other than 45S5 or S53P4.

### 7.1 Animal Experiments

Particle size was the focus of several in vivo studies, and one early study investigated 45S5 granule efficacy for bone regeneration in periodontal disease.^[^ ^90^ ^]^ When implanting 45S5 particles in the size ranges 90‐355, 500‐710, or 90‐710 µm for one month into surgically created defects in alveolar bone and root surfaces in patas monkeys (*Erythrocebus patas*), glass particles were found to be surrounded by connective tissue. For particles less than 500 µm in size, foamy macrophages and giant cells were detected in the connective tissue, but these were not observed for larger particles. Only the odd particle in one defect showed bone at its surface, while most particles were surrounded by adherent collagen fibers and phagocytes. With increasing implantation time, the number of particles with bone at their surface increased, and the number of phagocytes decreased. At nine months, foamy macrophages could still be detected in the defects, having been filled with the particle size range of 90–355 µm. Unfortunately, the publication contains a small number of figures only, not actually presenting results from different sizes, and we therefore have to rely on the information provided in the text.

In a later study, differences in size (45S5 granules in the size range of either 90 to 710 µm or 300 to 355 µm, implanted into drilled cylindrical cores 6 mm in diameter in the distal femoral metaphysis of New Zealand white rabbits) were studied in more detail.^[^ ^91^ ^]^ In each animal, the wide size range particles (90–710 µm) were implanted into the right limb, while the narrow range ones (300–355 µm) were implanted into the left limb. The average particle size of the wide size range was found to be significantly smaller than that of the narrow size range. The animals were injected intravenously with two fluorochrome compounds to label mineralizing bone, xylenol orange and calcein were injected 10 and 2 days, respectively, before sacrifice, and samples were analyzed histologically and biomechanically. At each time point, significantly more bone formed in sites implanted with the wide particle range than with the narrow one. The ratio of bone to graft was also significantly higher for the wide size range than for the narrow one. The authors explain these findings with more and smaller particles in the wide size distribution, resulting in a larger SA/V ratio compared to the narrow size distribution. As discussed above, an increase in SA/V ratio results in increased degradation and surface layer formation, thus probably providing more sites for osteoblast adhesion and osseous formation in vivo. For both size ranges and time points, apposition rate at the periphery of the defect was larger than at its center.^[^ ^91^ ^]^ This agrees with further findings by Zhang et al.,^[^ ^12^, ^24^ ^]^ where pH within the particle bed was much higher than in the surroundings, delaying apatite surface layer formation within the particle bed. Both a drastic pH increase and reduced CaP surface layer formation can be expected to reduce bone formation by osteoblasts, and this may thus explain the lower rate of bone formation in the center of the implanted granules.

Wheeler et al.'s^[^ ^91^ ^]^ key finding was, however, a decrease in particle size with increasing implantation time. The measured mean particle diameter decreased from 201 ± 96 µm (day 0) to 174 ± 17 µm (day 12; wide particle size range) and from 421 ± 185 µm (day 0) to 294 ± 27 µm (day 12, narrow particle size range). Interestingly, the rate of resorption showed no significant differences between the two particle size ranges used. No statistical differences between particle size ranges were observed during mechanical studies, i.e., when comparing peak compressive load, compressive stiffness, and compressive modulus during compressive strength tests of the cancellous grafts. Histological analyses showed the BG particles of either size range as amorphous granules surrounded by a bone matrix.^[^ ^91^ ^]^ No adverse cellular responses were observed, and newly formed bone within the grafted defects appeared to be normal. Osteogenic pouches, i.e., mesenchymal cells migrating within cracks to a protected central pouch within the bioactive particles, were observed in 4.5 (4 weeks) and 32.5% (12 weeks) of the wide particle size range (90‐710 µm) and in 6.0% (4 weeks) and 16.0% (12 weeks) of the narrow particle size range (300‐355 µm). At 12 weeks, significantly more osteogenic pouches were detected for the wide particle size range than for the narrow one. In addition, all pouches for the wide size range were ossified at 12 weeks, while ossification was observed in very few of the narrow particle size range. Pouches seemed to develop mostly in particles larger than 300 µm in diameter, but were observed in smaller particles, too. The authors concluded that a narrow particle range may adversely affect the quantity of osteointegration.

The studies by Wilson & Low^[^ ^90^ ^]^ and Wheeler et al.^[^ ^91^ ^]^ clearly demonstrate that BG implantation into bone defects can help to regenerate bone, with the BG degrading in the body over time to be replaced by natural bone. This discovery ultimately led surgeons to use 45S5 granules for bone regeneration rather than monoliths for bone replacement as in its early days of use.^[^ ^92^ ^]^

In vivo studies on osteoconduction in rabbits using S53P4 and two other BG compositions showed that granule size had an influence on bone growth from the surrounding tissue.^[^ ^93^ ^]^ While the authors investigated only one particle size range for S53P4, they studied two other BG (one of higher silica content and containing borate as an additional component, another one having a comparable silica content to S53P4 but also containing alumina) in two size ranges (200–250 and 630–800 µm). At both four and eight weeks, a mixture of the two particle size ranges showed significantly less bone formation than the larger particle size range alone for the alumina‐containing glass. For the borate‐containing glass, significantly less bone formation was observed at 4 weeks for the smaller size range compared to the larger one; however, at 8 weeks, no difference was apparent. The combined results by Wilson & Low,^[^ ^90^ ^]^ Wheeler et al.^[^ ^91^ ^]^ and Lindfors & Aho,^[^ ^93^ ^]^ demonstrate that BG particle size distribution, rather than just average (or median) particle size, is an important parameter to consider.

Interconnected pore size is thought to be a key parameter controlling cell ingrowth into 3D porous scaffolds. An in vivo study comparing BG porous scaffolds showed that, besides interconnected pore size, total porosity and strut dimensions are important parameters controlling degradation. Shi et al.^[^ ^94^ ^]^ prepared scaffolds from bioactive glass ICIE16, which was originally designed to improve high‐temperature processing without crystallization occurring.^[^ ^95^ ^]^ Scaffolds were prepared by either gel‐cast foaming or direct ink writing, and were investigated in vivo using the lateral femoral head defect rabbit model on New Zealand rabbits. µCT characterization showed that while surface areas were comparable within the error limits, foams showed larger specific surface area (176 vs 76 cm^2^ g^−1^), lower modal strut thickness (36 vs 167 µm), and larger porosity (75 vs 46%) compared to the 3D printed specimens. At ten weeks, the foam scaffold had nearly completely degraded, while the printed scaffold had not. At four weeks, more bone ingrowth was seen for the foams, but at ten weeks, printed scaffolds had surpassed them. These findings suggest that both foam and 3D printed scaffolds show certain advantages, and that a combination of both may help to optimize 3D porous scaffold structure for bone regeneration.

Results on these two types of scaffolds showed another interesting effect: new bone formation seemed to be particularly noticeable in concave pores of the foam and concave features of the printed scaffolds.^[^ ^94^ ^]^ This may suggest a topographical, i.e., physical, effect on bone formation; however, a chemical effect, such as increased ion concentration or advantageous surface energy in these areas, cannot be excluded.

An interesting series of studies, starting from material development and characterization via acellular immersion^[^ ^96^ ^]^ and in vitro cell culture experiments^[^ ^97^ ^]^ to in vivo studies^[^ ^98^ ^]^ showed that BG surface roughness may influence experimental outcome but that its precise effect is difficult to predict. Three melt‐derived BG compositions developed for improved high‐temperature processing without crystallization occurring were studied and included the well‐known composition 13‐93, but unfortunately neither 45S5 nor S53P4.^[^ ^99^ ^]^ BG granules were turned into microspheres by flame‐spraying, sintered, and either used directly or etched using a solution of 22 m NH_4_F and 8.5 M C8H_10_O_7_ to create a microrough surface. Analyses included surface roughness, mechanical testing, and compositional changes during etching. The latter revealed a depletion in modifier (i.e., metal) cations and phosphate in the etched surfaces, while surface topography showed the desired microroughness. Etched surfaces showed faster silica gel layer formation in contact with either SBF or Tris‐HCl buffer solution at early time points (up to six hours), while at later time points (up to 72 h) silica layer thickness was either the same or less than on control, i.e., unetched, surfaces. Adhesion of MG63 human osteoblast‐like cells was significantly enhanced on etched surfaces, while cell proliferation was not affected by etching. Subsequent in vivo studies in New Zealand white rabbits showed the effect of microroughness on new bone formation to depend on BG composition, with only one of the three compositions showing significantly enhanced new bone formation and bone affinity index, i.e., the fraction of bone in contact with the outer BG perimeter. These findings illustrate that an interplay between surface modification and BG composition may affect experimental outcomes.

### 7.2 Clinical Studies

A study on the clinical performance of S53P4 granules or plates in facial bone reconstruction highlighted the clinical relevance of the relationship between SA/V ratio and BG resorption. Suominen & Kinnunen^[^ ^100^ ^]^ included 13 patients in their study,^[^ ^100^ ^]^ eight men and five women. Indication for reconstruction varied and included orbital fracture, gunshot injuries, or craniotomy, among others. Granules (630–800 or 800–1000 µm in size) were implanted into 16 sites, e.g. subperiosteal pockets over frontal, temporal, zygomatic or maxillary bones or to obliterate frontal sinuses. Glass plates, which had been cut into a range of sizes from 8 x 10 to 15 x 29 mm and thicknesses of 1.5, 2.0, 2.5, or 3.0 mm, were used in orbital wall reconstruction and, in one patient, in maxillary augmentation. Such a wide variation in the type and site of defect limits statistical comparison, as pointed out by the authors. Results were evaluated by radiographs taken before and at one week and six months after surgery. In addition, quantitative computed tomography (CT) for bone mineral analysis and density measurement at the implant sites (presented in mg mL^−1^) was performed. Bone contact was evaluated and graded as good, moderate, or poor; autogenous bone was used as a control. Results showed the glass granules to resorb quickly. By contrast, no size change was observed for the plates. This finding agrees with the in vitro findings presented above, showing that a small SA/V ratio, as in the compact, non‐porous plates used here, delays degradation. The autogenous bone control resorbed at a rate similar to that of BG granules. CT further showed gaps, i.e., poor contact, between BG granules and host bone in 56% of the implantation sites. A decrease in density during the six‐month's follow‐up period suggests ingrowth of soft tissue. Implanted BG plates showed a small contact area only with the remaining orbital wall, but 85% of the plates were interpreted as showing either good or moderate bone contact. In 20%, osteoconductive bone growth was observed. As such, BG plate incorporation was judged as much better than that of BG granules or autogenous bone control. It is worth noting that the authors of this study investigated BG use for facial bone reconstruction, i.e., bone replacement, where BG resorption may negatively affect long‐term results, which is in contrast to BG use for bone regeneration, where resorption in timely accordance with bone formation is desired.

Clinical follow‐up over 14 years post‐implantation of S53P4 granules showed that complete degradation depends on defect size and possibly also particle size. The long‐term effects of S53P4 granules (BonAlive Biomaterials, Finland) for bone regeneration, i.e., as bone grafts in benign bone tumors, were investigated in 21 patients, with results being followed‐up, on average, over 14 years.^[^ ^88^ ^]^ Tumors were detected by radiography; their size (ellipsoidal volume) was estimated on both X‐rays and CT scans and classified as either large (mean 23.4/28.8 cm^3^; 64%) or small (2.3/1.1 cm^3^; 36%). Tumors were evacuated and the inner wall of bone refreshed using bone drills; afterwards, the cavity was filled with either S53P4 granules (eleven patients) or autogenous bone (ten patients). BG granule size varied with tumor size, with small tumor cavities being filled with particles in the size range of 1–2 mm and large cavities with granule sizes of 1‐2, 2‐3, or 3.15‐4 mm. During this follow‐up, implantation sites were examined using radiography, CT scans, and magnetic resonance imaging. In the group of small tumors, no BG granules were observed at the implantation site; however, for the large tumors, in six out of eight patients, ill‐defined glass granules were observed. Unfortunately, the authors do not distinguish different BG particle sizes in the large tumor group. Still, the study shows that, especially in large cavities, BG remnants may remain for a long time. Whether this was partially caused by some of the large cavities having been filled with larger particles, similar to what has been observed in vitro or in animal experiments, the authors did not investigate. However, considering the results by Zhang et al.,^[^ ^12^ ^]^ who showed that BG surface layer formation depended on the position of the particle within the particle bed, with particles at the center showing less layer formation and particularly less CaP formation than particles at the periphery, this finding of slower degradation in larger defects, is maybe not that surprising.

## 8 Conclusion

The rate at which reactions at the BG/solution interface occur during immersion can be controlled via the SA/V ratio, allowing for adjustment of ion release into solution, changes in osmotic pressure and pH, and the formation of surface layers such as an ion‐depleted silica gel layer and a surface layer of biomimetic apatite. For example, early BG implants intended to remain in the body permanently were made from cast glass pieces such as cones, while BG‐containing toothpaste, where a rapid reaction is required, contains fine BG powder with a large SA/V ratio and high reactivity.^[^ ^101^ ^]^ Experimental results show particle size distribution to be of more relevance here than average (or median) particle size. As BG reactivity with water is key also in its interaction with bacteria, cells, or tissue, BG morphology offers the possibility of controlling how fast body cells adhere, proliferate, or differentiate, or how easily bacteria adhere to a BG surface. SA/V ratio also has a direct influence on the rate at which BG degrades in the body. Ideally, degradation rate matches that of bone formation—but clinical studies have shown that in large defects, and especially when the SA/V ratio is small, the degradation of glass particles can be dramatically delayed, so that glass particles can still be found in the body years after implantation.

Besides morphology, contact between BG specimens and the surrounding solution, such as the position of the particles within the particle bed, has a pronounced influence on the surrounding pH and surface layer formation. As a consequence, the rate of bone formation in vivo varies between the center and the periphery of the implanted BG particles.

Another way to control how cells or bacteria interact with BG is through surface structuring. Depending on the size or orientation of surface features, cells adhere better and align or spread along the pattern—or bacteria are prevented from colonizing the surfaces, thus providing host cells with an advantage against microbes in the race for the surface.^[^ ^102^ ^]^ The combination of chemical and physical effects of surface functionalization, such as increased alkalinity combined with roughness, appears to be particularly promising for antimicrobial effects.

While the influence of the SA/V ratio on solubility behavior is well documented, there are very few studies on the influence of physical surface characteristics, especially on bacteria. There are also few studies comparing how the same morphology affects solubility, cells, and bacteria. Once in vitro studies provide us with better insight into these aspects, results can be verified in living systems.

## Conflict of Interest

The authors declare no conflict of interest.

## Supporting information



Supporting Information
