# Supplementary material for: Beyond Bioactive Glass Composition: Using Morphology to Improve In Vitro and In Vivo Performance
Source: Adv Healthc Mater. 2025 Sep 16;14(32):e02591. doi: 10.1002/adhm.202502591 (PMC12716176; doi:10.1002/adhm.202502591)
Supplement: Supplementary file 1 — Supporting Information [file ADHM-14-0-s001.pdf]

ADVANCED HEALTHCARE MATERIALS

Supporting Information

for *Adv. Healthcare Mater.*, DOI 10.1002/adhm.202502591

Beyond Bioactive Glass Composition: Using Morphology to Improve In Vitro and In Vivo Performance

*Meixin Su, Mert Ergin, Diana Horkavcová, Victoria Horbert, Georg Matziolis and Delia S. Brauer**

WILEY-VCH

Supporting Information

Beyond bioactive glass composition: Effects of morphology during *in vitro* and *in vivo* studies

*Meixin Su[1], Mert Ergin[2], Diana Horkavcová[3], Victoria Horbert[2], Georg Matziolis[2], Delia S. Brauer[1,]**

[1] Otto Schott Institute of Materials Research, Faculty of Chemistry and Earth Sciences, Friedrich Schiller University Jena, Lessingstr. 12 (AWZ), 07743 Jena, Germany

[2] Experimentelle Orthopädie, Universitätsklinikum Jena, Campus Eisenberg, Waldkliniken Eisenberg, Klosterlausnitzer Str. 81, 07607 Eisenberg, Germany

[3] Laboratory of Chemistry and Technology of Glasses, Department of Glass and Ceramics, University of Chemistry and Technology, Technická 5, 166 28 Prague 6, Czech Republic

E-mail: delia.brauer@uni-jena.de

Information on abbreviations used in the tables can be found in the Section Abbreviations towards the end of the document. Reference numbers refer to the reference list in this document.

Table S1. Summary of Bioglass 45S5 and BonAlive S53P4 morphology and immersion conditions during acellular immersion studies published in the literature in chronological order.

BG morphology & synthesis route	BG characterization	Immersion medium composition & starting pH	Immersion conditions & time points	Contact method & BG concentration & controls	Solution analyses	Sample & surface analyses	Reference
**45S5 particles:** < 5 µm (melt-derived, US Biomaterials)<br><br>**Comparison**: Variation of concentration	Particle size distribution, specific surface area, porosity, skeletal density, and textural features of the powders (details given in previous work[1])	SBF, see below,[2] pH 7.25	Orbital shaker shaking at 60 Hz, 37°C#<br><br>1, 2, 4, 22 h	Varying BG concentrations of 0.001, 0.0015, 0.002, 0.005, 0.010, or 0.015 g mL^{-1} corresponding to 0.05, 0.075, 0.10, 0.25, 0.50, or 0.75 g of BG in 50 mL<br><br>3 replicates	Obtained by filtration (1-µm paper); composition (ICP)	Filtered powder was rinsed with acetone & dried; analyzed by FTIR & XRD	Jones et al., 2001[3]
**45S5 particles**: 5-20 µm (fine), 90-300 µm (medium), 90-710 µm (coarse); (melt-derived; US Biomaterials)<br><br>**Comparison**: Variation of particle size Variation of solution composition	Particle size distribution, specific surface area, porosity, and morphological features (no details given)	Simulated body fluid: 7.996 g NaCl, 0.350 g $NaHCO_3$, 0.224 g KCl, 0.228 g $K_2HPO_4{\cdot}3H_2O$, 0.305 g $MgCl_2{\cdot}6H_2O$, 40 mL 1N-HCl, 0.278 g $CaCl_2$, 0.071 g Na_2SO_4, 6.057 g $NH_2C(CH_2OH)_3$ (Tris), pH 7.25<br><br>Cell culture medium: 400 mL a-MEM, 1.6 mL penicillin streptomycin,	Orbital shaker under 1 Hz at 37°C#<br><br>30 min, 1, 2, 4, 8, 17, and 22 h	0.5 g BG in 50 mL solution (i.e. 0.01 g mL^{-1})	Obtained by filtration; composition (ICP)	Filtered powder was rinsed with acetone & dried; analyzed by FTIR & XRD	Sepulveda et al., 2002[2]

		40 mL fetal calf serum, pH 7.4					
**45S5 sintered tape-cast discs**: tape-casting and sintering at 800, 900 or 1000°C (3 h) or 1000°C (6 h)<br><br>**Comparison**: sintering conditions	Crystal phases (XRD), structure (FTIR), flexural strength	SBF after Kokubo,[4] pH not provided<br><br>Tris buffer solution, concentration or pH not provided	Static, 37°C<br><br>1 hour to 8 weeks	Samples 11 mm Ø, 2 mm height were suspended in 25 mL or 200 mL of SBF or Tris	Compositional analysis (ICP-OES) of Tris solution	Samples soaked in acetone and allowed to dry<br><br>Structural changes (FTIR), surface morphology and composition (SEM with EDX), flexural strength	Clupper et al., 2001,[5] 2002[6] & Gough et al., 2004[7]
**45S5 fibers**: 20, 40 µm Ø, 2 cm length (MO-SCI Corporation)<br><br>**Comparison**: fiber diameter	No information given	SBF after Kokubo,[4] pH 7.25	Orbital shaker shaking at 175 rpm, 37°C	Surface area to volume ratio SA/V = 1.0 cm^{-1} (0.0266 g of 20 µm or 0.05323 g of 40 µm fibers in 20 mL)	None	Rinsing with acetone and drying at 60°C, analysis by FTIR and Raman spectroscopy	Clupper et al., 2004[8]
**45S5, S53P4 particles, fibers, plates**: particles 500-800 µm, fibers 20-500 µm Ø, plates 2 x 1.5 x 0.15 cm^3<br><br>**Comparison**: position of fibers (centre or outer surface of fiber bundle)	No details given	SBF after Kokubo,[4] pH 7.3	Static, 37°C<br><br>2-7 days	SA/V = 0.4 cm^{-1}	None	Rinsing with ultrapure water and ethanol, drying, embedding in resin, cross-sectioning and analysis by SEM with EDX	Zhang et al., 2007[9]
**45S5, S53P4 particles**: <45, 45-90, 90-250, 250-315, 315-500, 500-800, 800-1000 µm, glass plates (2 x	Calculation of surface area per 1.5 g of BG	SBF after Kokubo,[4] pH 7.3	Static, 37°C<br><br>continuous *in situ* pH measurements for 48 h	1.5 g BG in 15 mL (i.e. 0.01 g mL^{-1}); the BG was placed into a cavity at the bottom of the immersion container and the	Phosphate concentration quantified at 2, 4, 27 and 48 h for the finest fraction (S53P4 only)	Washing with ultrapure water and ethanol Particle samples at 48 h and plate samples at 72 h embedded in resin, cross-sectioned and analyzed by SEM with EDX	Zhang et al., 2008[10]

1.5 x 0.15 cm^3) as control (all melt-derived)<br><br>**Comparison**: particle size			control (discs): 4, 8, 24 or 72 h	tip of a micro pH electrode was placed among the particles glass discs (control) were immersed at 0.01 g mL and pH of solution measured using a conventional pH electrode			
**45S5 particles**: nanoscale particles 20-60 nm (nBG; flame-sprayed) microscale particles D_{50} 10 µm (µBG; melt-quenched)<br><br>**Comparison**: nBG *vs.* µBG	Specific surface area, morphology (TEM with EDX), composition (LA-ICP-MS), amorphous state (XRD), molecular structure (FTIR)	SBF after Kokubo,[11] pH 7.4 (at 37°C)	Orbital shaker shaking at 120 rpm, 37°C<br><br>4 h, 8 h, 1 day, 3 days and 7 days	75 mg BG powder in 50 mL solution	None	nBG powder was filtered, rinsed with distilled water, dehydrated with acetone and dried at 60°C for 24 h, analyzed by TEM with EDX, FTIR and XRD	Mačković et al., 2012[12]
**S53P4 microspheres**: 45-90, 90-125, 125-212, 300-500 µm (non-porous, flame-sprayed, BonAlive Biomaterials)<br><br>**Comparison**: microsphere size	Size distribution (laser diffraction), surface morphology (SEM) and composition (EDX)	SBF after Kokubo,[11] pH 7.4 (at 37°C) Tris-HCl (12 g tris-(hydroxymethyl)-aminomethane in 1600 mL H_2O), pH H 7.4 (at 37°C)	**Static**: 37°C: 24, 72, 168 h<br><br>**Dynamic**: 37°C, flow rate 0.2 mL min^{-1} (laminar flow): one week	**Static**: 0.4 cm^{-1} SA/V<br><br>**Dynamic**: 205 ± 5 mg microspheres (45–500 µm) in continuous flow-through reactor.	Composition (ICP-OES)	Composition (SEM with EDX), particle size distribution (laser diffraction) before and after experiments; calculation of normalized surface-specific mass loss rate	Sinitsyna et al., 2021[13]
**S53P4 particles, scaffolds**: 315-500 µm (melt-derived, BonAlive Biomaterials) as is or sintered into	**Scaffolds**: amorphous nature (XRD), porosity (SEM followed by image analysis),	SBF after Kokubo[4]<br><br>Tris-HCl, 50 mM, pH 7.4 (at 37°C)	**Dynamic**: 37°C, continuous flow 0.2 mL min^{-1}, 0-7 days	272 ± 5 mg scaffolds, 233 mg granules in continuous flow-through reactor.	Composition (ICP-OES)	Samples were washed with ultrapure water and ethanol, embedded in resin, cross-sectioned and analyzed by SEM with EDX	Aalto-Setälä et al., 2021[14]

scaffolds (60 min at 630°C; 54% porosity)<br><br>**Comparison**: granules vs. scaffolds	compressive strength			(weight difference due to granules packing being looser)			
**S53P4 microspheres**: 45-90, 90-125 µm (non-porous, flame-sprayed)<br><br>**Comparison**: microsphere size	Thermal properties, morphology (SEM), composition (EDX), size distribution (laser diffraction), amorphous state (XRD), molecular structure (FTIR)	SBF after Kokubo [11], pH 7.4 (at 37°C)<br>Tris-HCl (50 mM), pH 7.4 (at 37°C)	**Dynamic**: 37°C, flow rate 0.2 mL min^{-1} (laminar flow) up to 72 h	210 ± 5 mg microspheres in continuous flow-through reactor	Composition (ICP-OES)	Composition (SEM with EDX), molecular structure (FTIR), phase composition (XRD) at 8, 24, 72 h	Sinitsyna et al., 2022[15]
**45S5, S53P4 microspheres:** 125-200 µm, porous, flame-sprayed<br><br>**Comparison:** porosity	Morphology (SEM), composition (EDX), density & porosity (helium pycnometry), thermal properties (DSC)	Milli-Q® water, pH 7.8<br>SBF (after ISO 23317:2014), pH 7.4	**Milli-Q®**: Static, 37°C, 1-28 days<br>**SBF**: 37°C, 1-10 days, shaking at 120 r min^{-1}	**Milli-Q®**: 1% w/v<br>**SBF**: 75 mg in 50 mL	pH	Phase composition (XRD), mass loss, pH during immersion<br>Morphology (SEM) after immersion	Islam et al., 2022[16]

**45S5 or S53P4 granules, sintered scaffolds and crushed scaffolds:** granules 300-500 µm (melt-derived); porous and partially crystalline scaffold (sintered at 720°C (S53P4) or 1000°C (45S5) in N_2 for 90min)<br><br>**Comparison**: granules vs. scaffold vs. crushed scaffold	S53P4 scaffolds characterized previously[17]	SBF after Kokubo,[4] no information on pH provided<br><br>Tris-HCl (50 mM), pH 7.4 (at 37°C)	**Dynamic:** 37°C, flow rate 0.2 mL min^{-1} (laminar flow), 14 days for granules and crushed scaffolds; 21 days for sintered scaffolds	270-300 mg S53P4 granules and scaffolds; 195-230 mg 45S5 granules and scaffolds (smaller mass was used for preventing blockage in reactor tubes), continuous flow-through reactor	Composition (ICP-OES)	Samples rinsed with ethanol, dried, weighed and cast into epoxy resin, then polished and cross-sectioned; thickness and composition examined by SEM with EDX	Aalto-Setälä et al., 2023[18]
**S53P4 particles, microspheres**: 45-90 µm granules (melt-derived) or microspheres (flame-sprayed)<br><br>**Comparison**: granules vs. microspheres	Surface morphology (SEM), composition (EDX), size distribution (laser diffraction), specific surface area	Tris-HCl, 50 mM, (12 g tris-(hydroxymethyl)-aminomethane in 1600 mL deionized water); pH 7.4 (at 37°C)	Dynamic: 37°C, flow rate 0.2 mL min^{-1} (laminar flow) up to 24 h	200 mg of granules or microspheres weighed into the sample cell and Tris-HCl was fed through it	Composition (ICP-OES)	Samples dried at 40°C, washed, embedded in epoxy resin and polished using 3 polishing papers (600, 1000, 2500) to obtain cross sections; compositional analysis (SEM with EDX); calculation of mass loss; particle size distribution (laser diffraction); specific surface area at 4, 8, 24 h	Sinitsyna et al., 2024[19]

Quoted as stated in the original publications[2-3]

Table S2. Summary of Bioglass 45S5 and BonAlive S53P4 morphology and cell culture conditions during *in vitro* studies published in the literature in chronological order.

BG morphology & synthesis route	BG characterization	Sterilization & pre-conditioning	Cells & cell density	Culture medium composition	Culture conditions & time points	Contact method & BG concentration & controls	Analyses	Reference
**45S5 monoliths**: Discs 15 mm Ø, 3 mm height (US Biomaterials) polished using 800 and 1200 grit emery paper	No information given	**Sterilization**: dry heat at 180°C overnight<br><br>**Pre-conditioning**: cell culture medium, 72 h	Human primary osteoblasts<br><br>**Density**: 22,500 cells cm^{-2}	Dulbecco's modified Eagle's medium (DMEM) with 10% fetal bovine serum (FBS), 2 mM L-glutamine, 50 U mL^{-1} penicillin G, 50 µg mL^{-1} streptomycin B, 0.3 µg mL^{-1} amphotericin B	37°C, 95% air humidity, 5% CO_2<br><br>**Time points**: 2, 6, 12 days	Direct seeding onto discs or controls<br><br>SA/V not given<br><br>**Control**: TCP or Thermanox	Cell morphology + adhesion patterns (SEM), cell cycle (propidium iodide staining, flow cytometry), cell density, quantif-ication of apoptosis (fluorescent staining and fluor-escence microscopy), ECM formation (TEM and CSLM), collagen I, calcified nodule formation, ALP activity, osteo-calcin, protein content	Xynos et al., 2000a[20]
**45S5 particles**: 300-710 µm (US Biomaterials)	Compositional analysis (ICP) of culture medium with BG extracts	**Sterilization**: no information given<br><br>No pre-conditioning	Human primary osteoblasts<br><br>**Density**: 25,000 cells cm^{-2}	DMEM with 10% FBS, 2 mM L-glutamine, 50 U mL^{-1} penicillin G, 50 µg mL^{-1} streptomycin B, 0.3 µg mL^{-1} amphotericin B	37°C, 95% air humidity, 5% CO_2<br><br>**Time points**: various	Indirect<br><br>**BG extract**: incubating 1% w/v BG in DMEM for 24 h at 37°C. Particles removed by filtration through 0.20 µm filter before supplementing medium	**Compositional analysis (ICP-OES)** of culture medium (unconditioned and pre-conditioned)<br><br>**Cell analysis**: Extraction of RNA Analysis of gene expression using cDNA microarrays or RT-PCR	Xynos et al., 2000b[21]

						**Control**: Culture medium without BG extracts	Measurement of IGF-II protein	
**45S5 particles**: 300-710 µm (US Biomaterials)	Compositional analysis (ICP-OES) of culture medium with BG extracts	**Sterilization**: no information given<br><br>No pre-conditioning	Human primary osteoblasts<br><br>**Density**: no information given	DMEM with 10% FBS, 2 mM L-glutamine, 50 U mL^{-1} penicillin G, 50 µg mL^{-1} streptomycin B, 0.3 µg mL^{-1} amphotericin B	37°C, 95% air humidity, 5% CO_2<br><br>**Time point**: 48 hours	Indirect<br><br>**BG extract**: incubating 1% w/v BG in DMEM for 24 h at 37°C. Particles removed by filtration through 0.20 µm filter before supplementing medium<br><br>**Control**: Culture medium without BG extracts	**Compositional analysis (ICP-OES)** of culture medium (unconditioned and pre-conditioned)<br><br>**Cell analysis**: RNA extraction and gene expression analysis	Xynos et al., 2001[22]
**45S5 particles**: 90-150 µm (melt-derived, obtained from L. Hench)<br><br>Comparison: variation of pH, direct vs. indirect	No information given	**Sterilization**: samples were not sterilized<br><br>**Pre-conditioning**: soaking for up to 72 h in culture medium	Mouse osteoblasts, rat primary fibroblasts<br><br>**Density**: 15,650, 31,300, 62,600 cells cm^{-2}	**Osteoblasts:** α-MEM, 1% or 10% FBS, 2 mM 1-glutamine, 200 U mL^{-1} penicillin, 100 µg mL^{-1} streptomycin<br><br>**Fibroblasts**: DMEM with 1% or 10% FBS, 2 mM 1-glutamine, 200 U mL^{-1} penicillin, 100 µg mL^{-1} streptomycin	37°C, humidified air, 5% CO_2<br><br>**Time point**: 48 hours	Direct or indirect; glasses applied as suspensions (for indirect separated from cells by micro-porous membrane); adjustment of pH by adding Tris<br><br>BG concentration: 2.38 mg/cm^2<br><br>**Control**: soda lime silicate or borosilicate glass, positive control: 30% FBS	**Culture medium**: pH measurement<br><br>**Cells**: metabolic activity (lactate production), cell viability (MTT assay), intracellular Adenosine tri-phosphate, intra-cellular ions and membrane potential, cell proliferation *via* DNA synthesis, differentiation (ALP activity), protein quantification	Silver et al., 2001[23]
**45S5 particles**: 90-710 µm (US Biomaterials)	No information given	**Sterilization**: no information given	MG63	DMEM with 10% FBS, 50 µg mL^{-1} ascorbic acid,	37°C in humidified air with 5% CO_2	Direct seeding onto granules or control	Staining of sections of dehydrated cells	Hattar et al., 2002[24]

**Comparison**: pre-conditioned *vs.* non-preconditioned granules		**Pre-conditioning**: 0.2 M Tris buffer (pH 7.25) for 48 hours; non-preconditioned BG granules also used	**Density**: 20,000 cells cm^{-2}	10mM β-glycerophosphate, 50 U mL^{-1} penicillin/ streptomycin	**Time points**: 3, 5, 7, 10, 13 days	30 mg glass per 60 mm culture dish; no information given on volume of culture medium<br><br>**Control**: bio-inert 60S glass particles 90-710 µm	and imaging by light microscopy and TEM, quantification of protein content, RNA extraction and RT-PCR amplification, cytoenzymatic localization of ALP	
**45S5 monoliths**: Discs 15 mm Ø, 2 mm height, one side polished to 3 µm (US Biomaterials)<br><br>**Comparison**: polished *vs.* non-polished	Surface topography (optical interferometry, lateral resolution 1.12 µm)	**Sterilization**: no information given<br><br>**Pre-conditioning**: cell culture medium, 24 h	Human primary osteoblasts<br><br>**Density**: 80,000 cells cm^{-2}	DMEM with 10% FBS, 2% penicillin/ streptomycin, 0.85 mM ascorbic acid	37°C in humidified air with 5% CO_2<br><br>**Time points**: 15, 30, 60, 90 min, 4 h, 48 h	Direct seeding onto discs or controls<br><br>SA/V not given<br><br>**Control**: TCP or Thermanox discs	Actin cytoskeleton staining, degree of spreading and actin organization (Confocal microscopy), nodule formation (SEM), mineralization (alizarin red staining and Raman spectroscopy)	Gough et al., 2004[25]
**45S5 sintered tape-cast discs**: tape-casting and sintering at 800, 900 or 1000°C (3 h) or 1000°C (6 h)<br><br>**Comparison**: sintering conditions	BG characterization published by Clupper et al.[6]	No information on sterilization or pre-conditioning given	Human primary osteoblasts<br><br>**Density**: 40,000 cells cm^{-2}	DMEM with 10% FBS, 1% glutamine, 2% penicillin/ streptomycin, 50 µg mL^{-1} ascorbic acid-2 phosphate	37°C in humidified air with 5% CO_2<br><br>**Time points**: 90 min, 24 h	Direct and indirect<br><br>**BG extract**: cell culture medium, 24 h	Compositional analysis (ICP-OES) of culture medium with BG extracts<br><br>**BG**: Ion release and apatite form-ation in cell culture medium<br><br>**Cells**: attachment, apoptosis, necrosis (propidium iodide staining), nodule formation (alizarin	Gough et al., 2004[7]

							red), collagen I (propidium iodide, collagen-1 anti-body staining)	
**45S5 particles**, commercially obtained	Compositional analysis (ICP-OES) of conditioned culture medium before and after dilution	**Sterilization**: no information given<br><br>No pre-conditioning	Human fetal osteoblasts<br><br>**Density**: 10,000 cells cm^{-2}	DMEM-F12 NUT with 1% antibiotic/ antimycotic	37°C, 5% CO_2<br><br>**Time points**: 1, 3, 7, 14 days	Indirect<br><br>**BG extract**: 1% w/v BG in DMEM at 37°C overnight, diluted with DMEM-F12 to Si concentrations of 15-20 µg mL^{-1}<br><br>**Control**: Culture medium without BG extracts	**Compositional analysis (ICP-OES)** of pre-conditioned culture medium<br><br>**Spectroscopy**: Raman spectra were recorded of live cells at 37°C, followed by principal component and least squares spectral fitting analysis<br><br>**Protein expression**: Immunofluorescent staining for osteoblast phenotype and differentiation markers ALP, bone sialoprotein, collagen I; quantification by real-time RT-PCR	Jell et al., 2008[26]
**45S5 particles**: 300-710 µm (US Biomaterials)<br><br>**Comparison**: Si concentrations in	Compositional analysis (ICP-OES) of culture medium (unconditioned and pre-conditioned)	**Sterilization**: no information given<br><br>No pre-conditioning	Human fetal osteoblasts, human adult osteoblasts, human bone marrow	**Without osteogenic supplements**: DMEM-F12 NUT Mix HAM with 1% (v/v) FBS, 50 U mL^{-1}	37°C, 5% CO_2<br><br>**Time points**: 2, 4, 7, 14, 21 days	Indirect<br><br>**BG extract**: 1% w/v BG in DMEM at 37°C for 24 h. Particles removed by filtration through	**Compositional analysis (ICP-OES)** of culture medium (unconditioned and pre-conditioned)	Tsigkou et al., 2009[27]

culture medium 15 *vs*. 20 µg ml^{-1}			osteoprogenitor cells<br><br>**Density**: no information given	penicillin, 50 mg mL^{-1} streptomycin, 1% (v/v) L-glutamine<br>**With osteogenic supplements**: 10% FBS, 50 µg mL^{-1} β-glycerophosphate, 10 mM L-ascorbate-2-phosphate, 10^{-8} M dexamethasone		0.20 µm filter before supplementing medium, diluted with DMEM-F12 to Si concentrations of 15 or 20 µg mL^{-1}<br><br>**Control**: Culture medium without BG extracts	**Cell analysis**: Flow cytometry Live/dead viability/ cytotoxicity assay Real time RT-PCR analysis, ALP enzymatic activity, osteocalcin protein level, collagen I production, mineralization and bone nodule formation (Alizarin red staining)	
**45S5 particles**: nanoscale particles 20-60 nm (nBG; flame-sprayed) microscale particles D_{50} 10 µm (µBG; melt-quenched)<br><br>**Comparison**: nBG *vs*. µBG	Specific surface area, morphology (TEM with EDX), composition (LA-ICP-MS), amorphous state (XRD), molecular structure (FTIR)	**Sterilization**: Samples were dispersed in 70% ethanol and sonicated for 30-60 min, transferred into 48-well plates and dried in chamber<br><br>No pre-conditioning	MG63<br><br>**Density**: 100,000 cells mL^{-1}; final volume 600 µl	DMEM with 10% FBS, 1 vol% penicillin/ streptomycin	37°C in humidified air with 5% CO_2<br><br>**Time point**: 48 hours	Direct seeding onto particles<br><br>**BG concentration**: 0.1, 1, 10, 100, 200 µg mL^{-1}<br><br>**Control**: zinc oxide	**Light microscopy**: Cell distribution and morphology (light microscopy, CSLM), quantification of attached cells (lactate dehydrogenase activity), viability (mitochondrial activity), osteoblastic activity (specific ALP activity)	Mačković et al., 2012[12]
**45S5 surface structure**: melt-derived BG, structuring by soft lithography/ sintering (1050°C, 30 min): array of 100 µm squares in 100 µm distance; parallel stripes	Surface characterization, roughness (SEM and LSM ()	**Sterilization**: autoclaving, 121°C<br><br>**Pre-conditioning**: cell culture medium, 2 days	MG63 and rat mesenchymal cells (rMSCs)<br><br>**Density**: 100,000 cells mL^{-1}, 1 ml per sample	DMEM with 10% FBS, 1 vol% penicillin/ streptomycin	37°C in humidified air with 5% CO_2<br><br>**Time point**: 48 hours	Direct seeding onto surfaces<br><br>SA/V not given<br><br>**Control**: non-structured surface	Cell distribution, cytoskeleton formation, alignment and Spreading (elongation factor) by CSLM	Detsch et al., 2012[28]

separated by 50 µm wide channels, non-patterned surface (control)<br><br>Comparison: differently structured vs. non-structured surface								
**S53P4 particles**: 0.5-0.8, 1.0-2.0, 2.0-3.15 mm<br><br>Comparison: particle size, concentration	Light microscopy	No information given	CCD-18CO fibroblast cells<br><br>**Cell suspension**: 20.000 cells mL^{-2}	Eagle's minimal essential medium with 10% FBS, 1 % penicillin/ streptomycin	37°C, 5% CO_2<br><br>**Time points**: 24, 48, 72 h	**Direct:** BG concentration 0.01, 0.1, 1% w/v<br><br>Cells cultured in 75 cm^2 flask until confluence (approx. 14 d)	Cell proliferation (LDH), cell viability (WST-8), VEGF release (ELISA), light microscopy (cell morphology)	Detsch et al., 2014[29]
**S53P4 polished discs** granules (melt-derived, BonAlive Biomaterials), discs 5x5x1.5 mm^3 (melt-derived)<br><br>Comparison: presence of granules vs. absence of granules	No information given	No information given	Human primary osteogenic sarcoma SaOS-2 cells<br><br>**Density**: 2 × 10^4 cells per well	McCoy's 5A culture medium with 10% FBS, 1% penicillin/ streptomycin	37°C, 5% CO_2<br><br>normoxia (20.9% O_2; 0.35% CO_2)<br><br>hypoxia (6% O_2; 7% CO_2)<br><br>**Time points**: 4.5 h, 2, 4 days	Culture in the presence or absence of granules (200 mg mL^{-1})<br><br>Direct and indirect	pH, cell attach-ment, focal contacts (vinculin staining)	Pérez-Tanoira et al., 2015[30]
**45S5 surface structure**: melt-derived BG, (a) structuring by casting melt onto structured PtAu5 sheet: groove width 30 µm,	Surface characterization: SEM (cross-sections) and LSM (roughness)	**Sterilization**: 200°C for 2 hours<br><br>No information on pre-	MG63<br><br>**Density**: 20,000 cells cm^{-2}	No information on culture medium given	37°C, 5% CO_2<br><br>**Time point**: 24 hours	Direct seeding onto surfaces<br><br>SA/V not given<br><br>**Control**: polished surface and	Live/dead staining, fluorescence microscopy	Pföss et al., 2016[31]

ridge width 10 µm, groove depth 15 µm<br>(b) polished surface<br><br>**Comparision**: structured vs. non-structured surface (control)		conditioning given				borosilicate glass cover slip		
**45S5 surface structure**: melt-derived BG,<br>(a) structuring by casting melt onto structured PtAu5 sheet: groove width 30 µm, ridge width 10 µm, (a) groove depth 8 µm (str 8)<br>(b) groove depth 15 µm (str 15)<br>(c) polished surface (pol)<br><br>**Comparison**: differently structured vs. non-structured surface (control)	Amorphous state (XRD), surface topography, roughness (LSM), wettability (water contact angle)	**Sterilization**: 200°C for 2 hours<br><br>No information on pre-conditioning given	hMSCs and macrophages (osteoclasts) RAW264.7<br><br>**Density**:<br>**hMSCs**:<br>5,000 cells cm^{-2} (viability, immunostaining)<br>10,000 cells cm^{-2} (live/dead)<br>31,000 cells cm^{-2} (differentiation)<br>**Osteoclasts**:<br>1,500 cells cm^{-2}	**hMSCs** growth medium with 2% FBS, 1% penicillin/ streptomycin<br><br>**Osteoblasts**:<br>RAW medium (4.5 g L^{-1} DMEM high glucose, L-glutamine) with 10% FBS, 20 ml L^{-1} L-glutamine; medium without ("-") and with ("+") differentiation factors	37°C, 5% CO_2, 90% humidity<br><br>**Time points**:<br>**hMSCs**: 1, 3, 7, 10, 14, 21 days<br>**Osteoclasts**: 7, 14 days	Direct seeding onto surfaces<br><br>SA/V not given<br><br>**Control**: TCP	**hMSCs**:<br>Cell viability, live/dead analysis & orientation, immunostaining and fluorescence microscopy (shape and spatial relation between cells and scaffold), SEM (morphology), ALP activity (osteoblastic activity), Alizarin Red S staining (mineralization), protein quantification, DNA quantification (cell proliferation), quantitative real-time PCR (osteogenic gene expression)<br><br>**Osteoclasts**:<br>quantitative real-time PCR (osteoclastic gene	Höner et al., 2018[32]

							expression), two-photon LSM (visualization)	
**S53P4 polished discs** (melt-derived), surface polished, cleaned, silanized (APTES) and fibronectin-coated (10 µg mL^{-1})<br><br>**Comparison**: with vs. without surface silanization, fibronectin-coating	no information given	**Sterilization:** no information given	Mouse embryonic fibroblast cell line (MEF)<br><br>Cell density: no information given	High-glucose DMEM supplemented with 10% FBS, 1% penicillin/ streptomycin	37°C, 5% CO_2<br><br>Cell attachment for 2h at 37°C, followed by live cell imaging for 12h.	Direct seeding on discs<br><br>**Control:** Borosilicate glass coverslips	Cell immunostaining, & time-lapse imaging, cell shape, movement and division analyses, immunostaining & confocal florescence microscopy	Azizi et al., 2021[33]
**S53P4 particles:** 500-800 µm (melt-derived, BonAlive Biomaterials)<br><br>**Comparison**: BG concentration in culture medium	no information given	samples received sterile	HaCaT (immortalized human epidermal keratinocyte-derived cell line)<br><br>**Cell viability**: 161,000 cells mL^{-1}<br><br>**Scratch assay:** 59,900 cells mL^{-1}	DMEM with 10% FBS, 1% MEM non-essential amino acids, 0.5% penicillin/ streptomycin<br><br>Adult keratinocyte growth medium, 0.5% penicillin/ streptomycin	37°C, 5% CO_2<br><br>**Cell viability:** 48h initial cultivation then 2 days with BG-treated medium<br><br>**Scratch assay:** 48h before scratching, 48h afterwards	**Cell viability:** DMEM or KGM with 0.5, 1, 2.5, 5 or 10% (w/v) S53P4<br><br>**Scratch assay:** 0.9, 1.7 or 3.5% (w/v) S53P4 in direct contact	pH (at 6 days)<br><br>Cell viability (MTT, cell counting Kit-8), light microscopy, medium cytokine profile, statistics (Kruskal-Wallis)	Sarin et al., 2021[34]
**45S5, S53P4 microspheres:** 125-200 µm, porous, flame-sprayed<br><br>**Comparison:** porosity	Morphology (SEM), composition (EDX), density & porosity (helium pycnometry),	**Sterilization:** Washing twice in 70% ethanol for 15 mins each<br><br>**Pre-conditioning**:	3T3 mouse fibroblasts<br><br>**Indirect**: 8,000 cells cm^{-2} in 300 µL	DMEM with 10% FBS, 2% antibiotics–antimycotics, 1% L-Glutamine, 1% of non-essential amino acid, 2% HEPES buffer,	37°C, 5% CO_2, up to 7 days<br><br>**Time point**: 48 h in untreated medium, then in pre-	**Indirect**: 100 mg mL^{-1}<br><br>**Direct**: 10 mg BG in 300 µL	**Indirect**: metabolic activity (Alamar blue)<br><br>**Direct**: Imaging (light microscopy, SEM)	Islam et al., 2022[16]

	thermal properties (DSC)	37°C, 5% CO_2, for 48 h then replaced with fresh medium for up to 7 days, filtered sterile (0.22 µM)	**Direct**: 18,000 cells cm^{-2}	0.85 mM ascorbic acid	conditioned medium			
**S53P4 particles** 500-800 µm (melt-derived, MO-SCI, US) direct and BG-conditioned medium<br><br>**Comparison**: direct vs. indirect	Not provided	**Sterilization**: dry heat (180°C, 4 h)	Murine-derived RAW264.7 or J774-Dual macrophage-like cells<br><br>Cell density varied with type of experiment, e.g. 94,000 cells cm^{-2} in 80 µL medium or 50,000 cells cm^{-2} in 2 mL medium	DMEM, phenol-red containing, supplemented with heat-inactivated FBS (10%) and penicillin-streptomycin. J774-Dual culture medium further supplemented with further antibiotics (details cf. paper), for certain experiments also HEPES-buffered DMEM	37°C, 5% CO_2, culture medium changed every 2-3 days	Cells cultured in contact with BG or in BG-conditioned medium (15 mg mL^{-1}, 37°C, 5% CO_2, 48 h) for 24 h. Then cells were activated with bacterial lipopolysaccharide (10 ng L^{-1}) for 24 h; total culture time thus 48 h.<br><br>**Control**: Al_2O_3, 500 µm (direct or conditioned medium) and TCP	Oxygen consumption rate, extracellular acidification rate, RNA isolation and qPCR, nitrite generation, real-time live cell imaging for RAW264.7<br><br>interferon regulatory factor (IRF) and NF-κB pathways in J774-Dual	Kajander et al., 2025[35]

Table S3. Summary of Bioglass 45S5 or BonAlive S53P4 *in vitro* antimicrobial effects published in the literature in chronological order.

BG morphology & synthesis route	BG characterization	Sterilization	Bacteria & concentration	Culture medium	Culture conditions & time points	Contact method & BG concentration & controls	Analyses	Reference
**45S5, S53P4 particles:** 315-500 µm (S53P4: Abmin Technologies; 45S5: US Biomaterials)<br><br>**Comparison**: with vs. without serum pre-treatment	No information provided	Heat sterilization	*Haemophilus influenza* (NCTC K1157 type b), *Streptococcus pneumoniae* (ATCC 47186 type 3), radioactively labelled (^{3}H-thymidine solution)	Chocolate agar plate (*H. influenza*) and blood agar plate (*S. pneumonia*)	Granules with/without human serum preconditioning: 100 mg BG granules incubated with 1 mL diluted serum at room temperature for 10 min<br><br>Adhesion test: 30 – 60 min, 37°C	Direct contact, BG concentration 100 mg mL^{-1}	Adhesion: liquid scintillation counter	Stoor et al., 2001[36]
**45S5 discs:** melt-derived, surface patterned by femtosecond laser treatment[37]<br><br>**Comparison**: with vs. without surface patterning	surface roughness, wettability, chemical composition (including surface)	Autoclaving of medium at 121°C at 15 psi for 15 min<br><br>Samples wiped with 70% ethanol and air dried	*Staphylococcus aureus* (ATCC 6538P), *Pseudomonas aeruginosa* (ATCC 19154), *Escherichia coli* (K12 Strain BW25113)<br><br>5 x10^7 cells mL^{-1}	Bacterial growth medium prepared by dissolving yeast extract (0.5%), Peptone (0.5%) and sodium chloride (0.5%) in deionized water	Discs immersed in bacterial solution and incubated for 2 h, rinsed in sterile water, excess liquid was drained off	Imprints of the disc surface taken on nutrient agar plates and agar incubated for 24 h at 37°C	Growth of the adhered bacteria was observed visually and documented by a Kodak Gel logic imaging system revealing the adherence pattern	Shaikh et al., 2018[38]
**S53P4 particles:** 500-800, < 45 µm (BonAlive Biomaterials)	No information provided	Samples obtained packed sterile	*S. aureus* (ATCC 29213), *Staphylococcus epidermidis* (ATCC 35984), *Enterococcus faecalis* (ATCC	*S. aureus, S. epidermidis, E. faecalis, E. coli*: Müller Hinton broth	IMC: real-time for 24 h<br><br>CFU counting: 0, 6, 24, 48 h	IMC: ampoules containing BG (500-800 or < 45 µm) and medium at 400 or 800 mg mL^{-1}	Isothermal microcalorimetry (IMC), CFU counting	Gonzalez Moreno et al., 2020[39]

**Comparison**: particle size; concentration			19433), *E. coli* (ATCC 25922), *Candida albicans* (yeast, ATCC 90028)<br><br>5 x10^5 CFU mL^{-1}	*C. albicans*: RMPI 1640<br><br>CFU counting: Müller Hinton agar plates		CFU counting: 10-fold serial dilutions of sampled aliquots on agar plates<br><br>Controls: medium with microorganisms but without BG: positive (growth) control; medium without microorganisms: negative (sterility) control; medium containing BG only (without microorganisms): measurement of heat production by BG alone		
**45S5, S53P4 particles**: 32-125, 90-710, 500-710 µm, 1-2 mm<br><br>**Comparison**: particle size	No information provided	Packaged in heat-sealed pouches and sterilized by gamma irradiation (25 kGy)	*Streptococcus gordonii* (DL1, wild-type), *Veillonella parvula* (PK1910, wild-type), *P. aeruginosa* (PAO1, wound isolate), Methicillin-resistant Staphylococcus aureus (ATCC BAA-2313™, MRSA, wild-type)	*S. gordonii* grown in Brain Heart Infusion (BHI) broth, *V. parvula* cultured in BHI supplemented with 0.6% sodium lactate (BHIL), *P. aeruginosa* cultured in Luria-broth (LB) and MRSA cultured in nutrient broth (NB)	*S. gordonii* and *V. parvula* grown anaerobically at 37°C<br><br>*P. aeruginosa* and MRSA grown aerobically at 37 °C<br><br>**Time points**: *S. gordonii*, *V. parvula* and *P. aeruginosa* for 24 and 48 h,	**Antibacterial**: Overnight bacterial cultures centrifuged and resuspended with fresh corresponding media to an OD600 of 1.0, the resuspended bacterial cultures were diluted 1:1,000 into the media with pre-weighted glass particles (50 to 800 mg mL^{-1})	**Antibacterial effect**: optical density at 600 nm, quantitative<br>**Antibiofilm effect**: crystal violet staining, biomass at 562 nm<br>**Culture medium**: pH measurement (data not shown)	Zhou et al., 2022[40]

			**Density**: Bacterial culture with an initial OD600 of 1.0 diluted 1:1000 into media with pre-weighed BG particles.		MRSA for 24, 48 and 72 h	**Antibiofilm**: Bacteria inoculated at a 1:1,000 dilution in BHIL or TSB broth with 0.5% yeast extract and 0.5% glucose, glass particles were aliquoted with 24 h established biofilms with final concentration of 100-200 mg mL^{-1}<br><br>**Control**: Bacterial cells in broth without treatment		

Table S4. Summary of Bioglass 45S5 or BonAlive S53P4 morphology and experimental conditions during *in vivo* studies published in the literature in chronological order.

BG morphology & synthesis route	Type of experiment (species)	Defect & implantation site	Control material	Analyses	Reference
**45S5 particles**:<br>90-355, 500-710, 90-710 µm (melt-derived)<br><br>**Comparison**: particle size range	Animal experiment (patas monkey*)	Periodontal<br><br>Alveolar bone, root surfaces	Hydroxyapatite, tricalcium phosphate	Histology	Wilson & Low, 1992[41]
**45S5 particles**:<br>90-710 µm (melt-derived, US Biomaterials); 300-360 µm (melt-derived, Orthovita)<br><br>**Comparison**: particle size range	Animal experiment (New Zealand white rabbit)	Cancellous bone<br><br>Distal femoral metaphysis	Normal bone	Histology, biomechanics	Wheeler et al., 1998[42]
**S53P4 particles, discs**<br><br>**Comparison**: granules vs. discs	Clinical study	Various: 36 sites in 13 patients	Parietal bone	Clinical examination, radiographs, CT	Suominen & Kinnunen, 1996[43]
**S53P4 particles**<br>and two other compositions, 200-250, 630-800 µm (melt-derived)<br><br>**Comparison**: composition, particle size range	Animal experiment (rabbit)	Distal femur from fovea intercondylaris to cortex	None	Histology	Lindfors & Aho, 2003[44]
**S53P4 particles**<br>1-2, 2-3, 3.15-4 mm (melt-derived, BonAlive Biomaterials)<br><br>**Comparison**: defect size, particle size range	Clinical study	Benign tumors (various)<br><br>Various sites (e.g. proximal humerus, distal tibia, finger)	Autogenous bone	Radiographs, CT for tumor volume and follow-up examinations, patient questionnaire, MRI<br>Statistics: ANOVA	Lindfors et al., 2010[45]

* Erythrocebus patas; in the original publication[41] referred to as patus monkey, Erythrocebus patus [sic].

Abbreviations

α-MEM – Alpha-modified Eagle's minimum essential medium
AES – Auger electron spectroscopy
ALP – alkaline phosphatase
APTES – (3-Aminopropyl)triethoxysilane
BG – bioactive glass
BSA – bovine serum albumin
CSLM – confocal scanning laser microscopy
CT – computed tomogram
DMEM – Dulbecco's Modified Eagle's Medium
ECM – extracellular matrix
EDX – energy dispersive X-ray analysis
FBS – fetal bovine serum
FTIR – Fourier-transform infrared spectroscopy
HA – hydroxyapatite
HEPES – 4-(2-hydroxyethyl)-1-piperazineethanesulfonic acid
hMSCs – human mesenchymal stromal cells
IMC – isothermal microcalorimetry
ICP-OES – inductively coupled plasma optical emission spectroscopy
LA-ICP-MS – laser ablation inductively coupled plasma mass spectroscopy
LSM – laser scanning microscopy
MRI – magnetic resonance imaging
mRNA – messenger RNA
Ø – diameter
qPCR – quantitative polymerase chain reaction
PBS – phosphate buffered saline
rpm – revolutions per minute
RT-PCR - reverse transcription polymerase chain reaction
SA/V – surface area per volume ratio
SEM – scanning electron microscopy
TCP – tissue culture plastic
TEM – transmission electron microscopy
Tris – tris(hydroxy) methylaminomethane
w/V – weight per volume
XPS – X-ray photoelectron spectroscopy
XRD – X-ray diffraction

References

[1] P. Sepulveda, J. R. Jones, L. L. Hench, *J Biomed Mater Res* **2001**, *58*, 734-740. https://doi.org/10.1002/jbm.10026.
[2] P. Sepulveda, J. R. Jones, L. L. Hench, *J Biomed Mater Res* **2002**, *61*, 301-311. https://doi.org/10.1002/jbm.10207.
[3] J. R. Jones, P. Sepulveda, L. L. Hench, *J Biomed Mater Res* **2001**, *58*, 720-726. https://doi.org/10.1002/jbm.10053.
[4] T. Kokubo, H. Kushitani, S. Sakka, T. Kitsugi, T. Yamamuro, *J Biomed Mater Res* **1990**, *24*, 721-734. https://doi.org/10.1002/jbm.820240607.
[5] D. C. Clupper, J. J. Mecholsky, Jr., G. P. LaTorre, D. C. Greenspan, *J Biomed Mater Res* **2001**, *57*, 532-540. https://doi.org/10.1002/1097-4636(20011215)57:4<532::aid-jbm1199>3.0.co;2-3.
[6] D. C. Clupper, J. J. Mecholsky, G. P. LaTorre, D. C. Greenspan, *Biomaterials* **2002**, *23*, 2599-2606. https://doi.org/10.1016/S0142-9612(01)00398-2.
[7] J. E. Gough, D. C. Clupper, L. L. Hench, *J Biomed Mater Res A* **2004**, *69A*, 621-628. https://doi.org/10.1002/jbm.a.30024.
[8] D. C. Clupper, J. E. Gough, P. M. Embanga, I. Notingher, L. L. Hench, M. M. Hall, *J Mater Sci-Mater M* **2004**, *15*, 803-808. https://doi.org/10.1023/B:JMSM.0000032821.32577.fc.
[9] D. Zhang, H. Arstila, E. Vedel, H. O. Ylänen, L. Hupa, M. Hupa, *Key Eng Mat* **2007**, *361-363*, 225-228. https://doi.org/10.4028/www.scientific.net/KEM.361-363.225.
[10] D. Zhang, M. Hupa, L. Hupa, *Acta Biomater* **2008**, *4*, 1498-1505. https://doi.org/10.1016/j.actbio.2008.04.007.
[11] T. Kokubo, H. Takadama, *Biomaterials* **2006**, *27*, 2907-2915.
[12] M. Mačković, A. Hoppe, R. Detsch, D. Mohn, W. J. Stark, E. Spiecker, A. R. Boccaccini, *J Nanopart Res* **2012**, *14*. https://doi.org/10.1007/s11051-012-0966-6.
[13] P. Sinitsyna, O. Karlström, L. Hupa, *J Am Ceram Soc* **2021**, *105*, 1658-1670. https://doi.org/10.1111/jace.18014.
[14] L. Aalto-Setälä, P. Uppstu, P. Sinitsyna, N. C. Lindfors, L. Hupa, *Materials* **2021**, *14*. https://doi.org/10.3390/ma14174834.
[15] P. Sinitsyna, O. Karlström, C. Sevonius, L. Hupa, *J Non-Cryst Solids* **2022**, *591*. https://doi.org/10.1016/j.jnoncrysol.2022.121736.
[16] M. T. Islam, N. A. Nuzulia, L. Macri-Pellizzeri, F. Nigar, Y. W. Sari, I. Ahmed, *J Biomater Appl* **2022**, *36*, 1427-1443. https://doi.org/10.1177/08853282211059294.
[17] S. Fagerlund, J. Massera, N. Moritz, L. Hupa, M. Hupa, *Acta Biomater* **2012**, *8*, 2331-2339. https://doi.org/10.1016/j.actbio.2012.03.011.
[18] L. Aalto-Setälä, M. Siekkinen, N. Lindfors, L. Hupa, *Biomedical Materials & Devices* **2023**, *1*, 871-881. https://doi.org/10.1007/s44174-022-00059-4.
[19] P. Sinitsyna, M. Engblom, L. Hupa, *J Non-Cryst Solids* **2024**, *637*. https://doi.org/10.1016/j.jnoncrysol.2024.123029.
[20] I. D. Xynos, M. V. J. Hukkanen, J. J. Batten, L. D. Buttery, L. L. Hench, J. M. Polak, *Calcified Tissue Int* **2000**, *67*, 321-329. https://doi.org/10.1007/s002230001134.
[21] I. D. Xynos, A. J. Edgar, L. D. K. Buttery, L. L. Hench, J. M. Polak, *Biochem Bioph Res Co* **2000**, *276*, 461-465. https://doi.org/10.1006/bbrc.2000.3503.
[22] I. D. Xynos, A. J. Edgar, L. D. K. Buttery, L. L. Hench, J. M. Polak, *J Biomed Mater Res* **2001**, *55*, 151-157. https://doi.org/10.1002/1097-4636(200105)55:2<151::Aid-Jbm1001>3.3.Co;2-4.
[23] I. A. Silver, J. Deas, M. Erecinska, *Biomaterials* **2001**, *22*, 175-185. https://doi.org/10.1016/S0142-9612(00)00173-3.
[24] S. Hattar, A. Berdal, A. Asselin, S. Loty, D. C. Greenspan, J. M. Sautier, *Eur Cells Mater* **2002**, *4*, 61-69. https://doi.org/10.22203/eCM.v004a05.
[25] J. E. Gough, I. Notingher, L. L. Hench, *J Biomed Mater Res A* **2004**, *68A*, 640-650. https://doi.org/10.1002/jbm.a.20075
[26] G. Jell, I. Notingher, O. Tsigkou, P. Notingher, J. M. Polak, L. L. Hench, M. M. Stevens, *J Biomed Mater Res A* **2008**, *86A*, 31-40. https://doi.org/10.1002/Jbm.A.31542.
[27] O. Tsigkou, J. R. Jones, J. M. Polak, M. M. Stevens, *Biomaterials* **2009**, *30*, 3542-3550. https://doi.org/10.1016/J.Biomaterials.2009.03.019.
[28] R. Detsch, O. Guillon, L. Wondraczek, A. R. Boccaccini, *Adv Eng Mater* **2012**, *14*, B38-B44. https://doi.org/10.1002/Adem.201180068.
[29] R. Detsch, P. Stoor, A. Grunewald, J. A. Roether, N. C. Lindfors, A. R. Boccaccini, *J Biomed Mater Res A* **2014**, *102*, 4055-4061. https://doi.org/10.1002/jbm.a.35069.
[30] R. Pérez-Tanoira, T. J. Kinnari, T. Hyyrynen, A. Soininen, L. Pietola, V. M. Tiainen, Y. T. Konttinen, A. A. Aarnisalo, *J Mater Sci-Mater M* **2015**, *26*, 246. https://doi.org/10.1007/s10856-015-5568-2.
[31] B. Pföss, M. Höner, M. Wirth, A. Bührig-Polaczek, H. Fischer, R. Conradt, *Biomed Glasses* **2016**, *2*, 63-71. https://doi.org/10.1515/bglass-2016-0008.
[32] M. Höner, I. Lauria, C. Dabhi, S. Kant, R. E. Leube, H. Fischer, *J Biomed Mater Res A* **2018**, *106*, 1965-1978. https://doi.org/10.1002/jbm.a.36399.
[33] L. Azizi, P. Turkki, N. Huynh, J. M. Massera, V. P. Hytönen, *ACS Omega* **2021**, *6*, 22635-22642. https://doi.org/10.1021/acsomega.1c02669.

[34] J. Sarin, M. Vuorenmaa, P. K. Vallittu, R. Grenman, P. Bostrom, P. Riihila, L. Nissinen, V. M. Kahari, J. Pulkkinen, *Otology & Neurotology* **2021**, *42*, e559-e567. https://doi.org/10.1097/MAO.0000000000003057.

[35] K. Kajander, N. Nowak, N. Vaziri, P. K. Vallittu, T. J. Heino, J. A. Määttä, *J Mater Sci-Mater M* **2025**, *36*, 13. https://doi.org/10.1007/s10856-025-06861-y.

[36] P. Stoor, E. Söderling, R. Grénman, *J Biomed Mater Res B* **2001**, *58*, 113-120. https://doi.org/10.1002/1097-4636(2001)58:1<113::aid-jbm170>3.0.co;2-v.

[37] K. Sharma, S. Kedia, A. K. Singh, C. B. Basak, A. K. Chauhan, S. Basu, S. Sinha, *J Non-Cryst Solids* **2016**, *440*, 43-48. https://doi.org/10.1016/j.jnoncrysol.2016.02.012.

[38] S. Shaikh, D. Singh, M. Subramanian, S. Kedia, A. K. Singh, K. Singh, N. Gupta, S. Sinha, *J Non-Cryst Solids* **2018**, *482*, 63-72. https://doi.org/10.1016/j.jnoncrysol.2017.12.019.

[39] M. Gonzalez Moreno, M. E. Butini, E. M. Maiolo, L. Sessa, A. Trampuz, *Colloids and Surfaces B: Biointerfaces* **2020**, *189*, 110853. https://doi.org/10.1016/j.colsurfb.2020.110853.

[40] P. Zhou, B. L. Garcia, G. A. Kotsakis, *BMC Microbiology* **2022**, *22*, 212. https://doi.org/10.1186/s12866-022-02617-8.

[41] J. Wilson, S. B. Low, *J Appl Biomater* **1992**, *3*, 123-129. https://doi.org/10.1002/jab.770030208.

[42] D. L. Wheeler, K. E. Stokes, R. G. Hoellrich, D. L. Chamberland, S. W. McLoughlin, *J Biomed Mater Res* **1998**, *41*, 527-533. https://doi.org/10.1002/(sici)1097-4636(19980915)41:4<527::aid-jbm3>3.0.co;2-e.

[43] E. Suominen, J. Kinnunen, *Scandinavian Journal of Plastic and Reconstructive Surgery and Hand Surgery* **1996**, *30*, 281-289. https://doi.org/10.3109/02844319609056406.

[44] N. C. Lindfors, A. J. Aho, *J Mater Sci-Mater M* **2003**, *14*, 365-372. https://doi.org/10.1023/A:1022988117526.

[45] N. C. Lindfors, I. Koski, J. T. Heikkila, K. Mattila, A. J. Aho, *J Biomed Mater Res B* **2010**, *94B*, 157-164. https://doi.org/10.1002/Jbm.B.31636.
